# The backfiring effect of weak AI safety regulation

**DOI:** 10.1073/pnas.2509768123

**Published:** 2026-07-20

**Authors:** Benjamin Laufer, Jon Kleinberg, Hoda Heidari

**Affiliations:** ^a^ Department of Information Science, Cornell Tech, New York, NY 10044; ^b^ Department of Computer Science and Information Science, Cornell University, Ithaca, NY 14853; ^c^ Machine Learning Department and the Institute for Software, Systems, and Society, Carnegie Mellon University, Pittsburgh, PA 15213

**Keywords:** AI Regulation, General-purpose AI, AI Safety, Game theory

## Abstract

Recent policy proposals aim to improve the safety of general-purpose AI, but there is little understanding of the efficacy of different regulatory approaches. We present a strategic model that explores interactions between safety regulation, general-purpose AI technology creators, and domain specialists—those who adapt the technology for specific applications. Our analysis examines how regulatory measures targeting different parts of the AI development chain affect the outcome of this game. Our model assumes AI technology is characterized by two key attributes: safety and performance. The regulator first sets a minimum safety requirement that applies to one or both players. The general-purpose creator then invests in the technology, establishing its initial safety and performance levels. Next, domain specialists refine the AI for their use cases, updating the safety and performance levels and taking the product to market. Resulting revenue is shared between the specialist and generalist. Our analysis reveals two insights: first, weak safety regulation imposed predominantly on domain specialists can backfire. While it might seem logical to regulate AI use cases, our analysis shows that weak regulations targeting domain specialists alone can reduce safety in a large class of parameterizations. Second, in contrast to the previous finding, we observe that stronger, well-placed regulation can mutually benefit all players. When regulators impose appropriate safety standards on both general-purpose AI creators and domain specialists, the regulation can function as a commitment device, leading to safety and performance gains, surpassing what is achievable under no regulation or regulating only one player.

## Significance

Proposed regulatory approaches to promote safe development of AI differentiate between entities developing base models and entities adapting the technology for a particular domain. We develop a formal approach to reasoning about how such regulations affect these entities’ incentives to invest in AI safety and performance. Our analysis reveals counterintuitive phenomena: if minimum requirements on safety investments are improperly formulated, they can inadvertently lower overall safety because upstream actors ‘free-ride’ on downstream mandates. In contrast, other, better-aligned regulations not only improve safety but also unlock strategy profiles that raise both parties’ utilities. The work demonstrates an application of game theory to reasoning about incentives in AI development and the possible effects of alternative regulatory interventions.

As Generative AI and related technologies gain traction, there is an increasing number of proposals for regulation to improve safety. Many of these proposals must at some level grapple with the following question: Who should be targeted with AI regulation—the producers of general-purpose AI models^*^ or the domain-specialists who adapt the technology for specific use cases? There are seemingly reasonable positions that favor regulating one entity, the other, both, or neither. For example, the downstream domain specialists and deployers are some of the last entities to exert influence on the technology before it interacts with consumers directly, so it is perhaps reasonable that regulation for consumer safety might target requirements at these entities. In contrast, the upstream entities developing general-purpose models exert impact on these models earlier in their development trajectories, facilitating or hindering downstream adoption, which might justify certain regulatory requirements including disclosure mandates (3) and liability standards. Of course, even regulations that solely target one of these actors might impact the other, because their incentives and decisions are intertwined.

We have seen variants of these debates play out as different jurisdictions and policymakers have proposed various regulatory approaches to AI. A number of existing regulation proposals leverage the observation that AI is developed by multiple interacting actors. Examples include Colorado’s AI Act, California’s Senate Bill 1047, and the EU AI Act. These frameworks attempt to define the relevant actors, such as base developers and downstream deployers, in order to design conditions and stipulations for determining whether and to whom liability standards, disclosure requirements, or other interventions apply. These conditions and stipulations vary across proposals and policies, with possibly significant implications for the incentives of the players involved in the development of AI technologies and applications.

## Modeling the Impact of Regulatory Regimes on AI Performance and Safety.

Given that there are a range of different possible approaches to targeting AI regulation and assessing the impact of each alternative empirically is prohibitive, formal models can enable reasoning about the various regulatory impacts, with the caveat that further empirical observations of regulatory effects are important for challenging, iterating, and informing modeling approaches. This paper puts forward a strategic model of the interactions between a general-purpose technology producer (*G*) and a domain specialist (*D*), building on the “fine-tuning games” model proposed by Laufer et al. (4). As the two actors develop an AI technology, they each decide whether and how to invest in two key attributes of technology: performance, denoted by *α*, and safety, denoted by *β*. We assume these actors are operating in a market; each actor experiences some cost for their investment in safety and performance, and obtains a share of the revenue out of the deployment of the AI product/service in the market.

For the sake of our discussion, safety refers to investments that mitigate either the probability or the severity of harm from the development or deployment of an AI system. Harm is accordingly the product of i) the likelihood of misuse or hazardous failure and ii) the severity of such misuse or failure. Examples of safety investments include market research into unsafe uses, product-driven filters, and posttraining procedures that prevent unsafe, harmful, hateful, or other outputs. Performance refers to investments leading to domain-specific fitness, including capabilities for domain-specific tasks. These might be advanced by general capabilities (e.g., reasoning, coding, multilingual support) or through domain-specific fine-tuning (e.g., medical notes summarization efficacy). We note that though we name the technology’s attributes this way, the model we will introduce could be adapted to reason about other possibly interrelated attributes that are demanded by consumers, where one is being regulated.

**Fig. 1.**
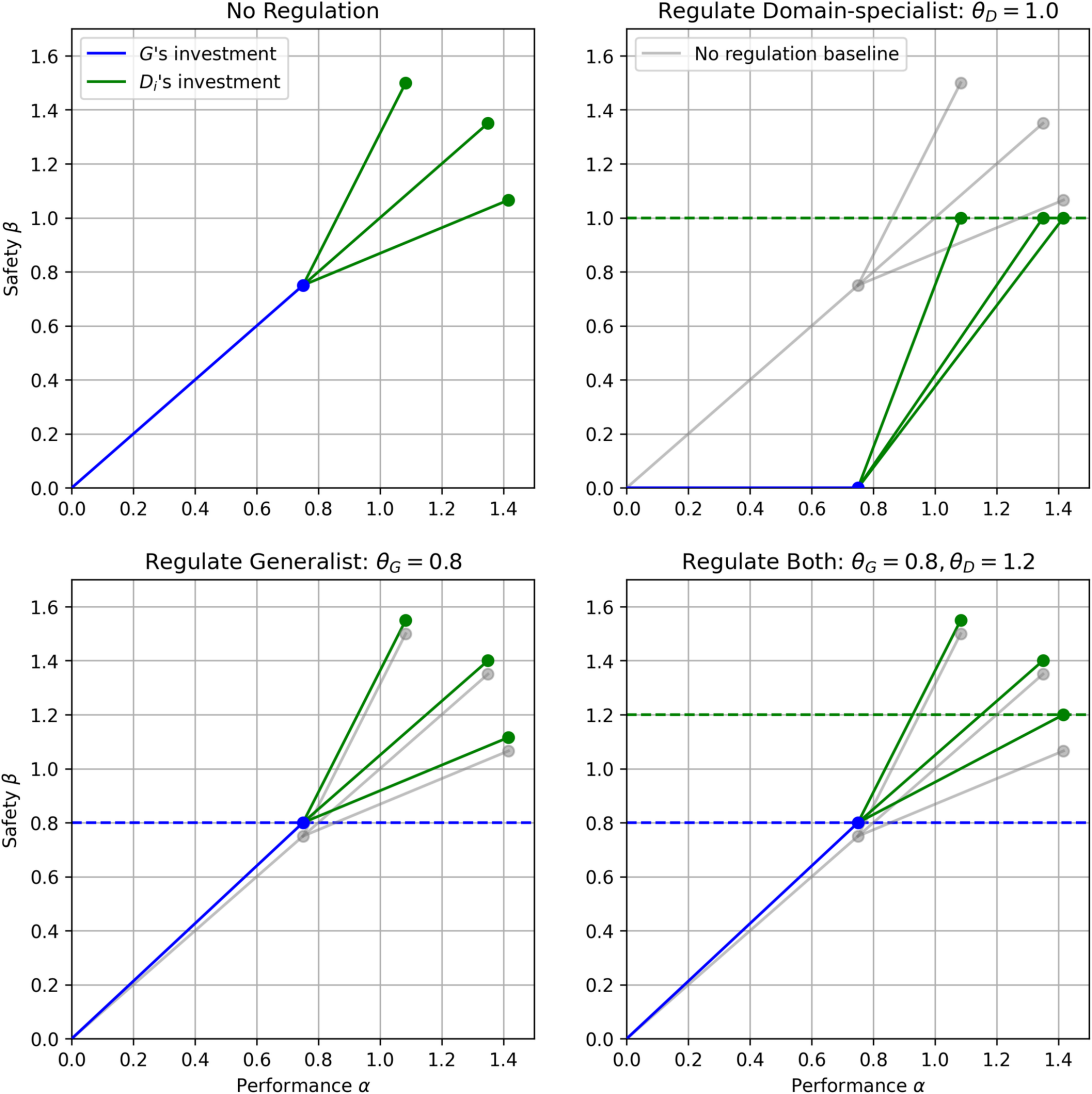
Illustrative example of our game-theoretic model. This numeric instance of the game consists of one general-purpose producer and three domain-specialists. Each player has a different utility in performance-safety space which dictates the path of development. Within this setting, the no-regulation game (*Upper Left*) reveals the players’ investment efforts when no floor is imposed on safety. Regulating the domain-specialist alone (*Upper Right*) exhibits backfiring for all three domains, meaning the regulated safety level is lower than it would be without regulation. In this particular example, the same floor is assumed for all three domain-specialists. Regulating the generalist alone (*Lower Left*) improves the safety level slightly across all three domains, compared to no-regulation. Finally, a regime that targets both generalists and specialists with regulation (*Lower Right*) is able to 1) retain the improved safety performance from regulating the generalist, 2) improve the safety level of least-safe domain-specialist, while 3) avoiding backfiring. The purpose of this figure is to visualize the model’s incentive mechanisms; none of these panels represent real regulations.

To provide some intuition for what the investment pattern in these attributes might look like, consider the following hypothetical example. A large internet technology firm *G* is investing significant capital toward producing a multipurpose language model, trained to generate text in response to user prompts. In this example, the language model has potential uses in three distinct domains—say, by healthcare providers ($D_{1}$), law firms ($D_{2}$), and financial services ($D_{3}$). The general-purpose developer moves first, and in light of the particular costs she faces and the anticipated responses from the downstream players, she chooses a certain strategy, represented by a pairing of performance and safety investments $\left(\alpha_{0},\beta_{0}\right)$. Once the investment by the general-purpose developer has been made, the attributes of the technology at this stage can be thought of as akin to a “base camp,” from which domain specialists may choose to climb further by investing their own effort toward improving the technology’s safety and/or performance in their respective domains. Of course, each domain faces their own delicate balance of safety risks and performance costs, so the ultimate safety and performance pairs $\left(\alpha_{i},\beta_{i}\right)$ (i=1,2,3) differ across the three domains. See Fig. 1 (*Upper Left*) for a visualization of the investment decisions made by *G*, $D_{1}$, $D_{2}$, and $D_{3}$.

Equipped with this intuition about how these actors behave in an unregulated market, we now turn to our notion of regulation. We conceive of regulation as shaping the game in which players choose their strategies. In particular, this paper will focus specifically on safety regulation. Our formal model assumes regulation imposes a constraint in the form of a lower bound on the players’ choice of safety investment (i.e., $\beta_{i}$’s). If a player does not meet the regulatory lower bound on safety, they will be penalized. This regulatory regime can be described using two parameters $\left(\theta_{G},\theta_{D}\right)$, representing the set of thresholds constraining the strategy space of *G* and *D*, respectively. The regulation can target the domain-specialist only ($\theta_{G}=0,\theta_{D}>0$), the generalist only ($\theta_{G}=\theta_{D}>0$), both players ($\theta_{D}>\theta_{G}>0$), or neither ($\theta_{G}=\theta_{D}=0$). In addition to the decision of who to target, the regulation encodes a decision about what level to set the safety standards. Smaller values of *θ* are less costly to comply with, and hence capture weaker safety requirements.

Before summarizing our findings, we caveat that our model and results are theoretical in nature: they characterize equilibrium behavior in a stylized game of structured incentives and decisions, with the goal of establishing existence results about when certain outcomes and strategies can arise. The figures in the introduction and numerical examples throughout the paper are illustrations representing instantiations of the model, not calibrated measurements of contemporary regulation. Our formal results define behaviors and characterize classes of parameter values for which these behaviors occur. Our findings should not be read as empirical observations about the current state of AI policy, rather they should be understood as characterizing the range of possibilities. Further discussion on how to interpret the model appears just after the model’s introduction (“uses for the conceptual model”).

## First Insight: Weak Safety Regulation Can Backfire.

Turning back to our illustrative hypothetical depicted in Fig. 1, we observe that the model can exhibit a striking incentive effect in the second panel, which depicts a scenario where regulation is targeted at the domain-specialist. In this scenario, the safety investment has worsened. How could our notion of safety regulation—a simple floor dictating a minimum investment level—lead to a less safe product? The mechanism leading to this phenomenon arises because the generalist *G* is aware of the regulatory safety requirements imposed on domain-specialists, and can use it to her advantage. When the regulator requires that a technology meets a certain level of safety investment by the time it reaches the market, the generalist has an opportunity to engage in a sort of free-riding behavior. The generalist is comfortable setting up the base camp at lower altitude, because she knows that the domain-specialist nonetheless has to climb to a level of investment that complies with regulation.^†^

The scenario described above is a worked example of a more general phenomenon in our model, which we describe as regulatory backfiring. A safety regulation backfires if it yields a total investment in safety lower than the safety investment achieved with no regulation. We identify a number of properties of this phenomenon—for example, backfiring only occurs when the regulation is weak, meaning the floor on safety is at or below the level reached in the absence of regulation. Backfiring can occur when *D* is targeted with regulation or when both *G* and *D* are targeted with regulation, but does not occur when only *G* is targeted. Backfiring only occurs in scenarios where the domain-specialist minimally complies with the safety regulation, meaning that the magnitude of backfiring is given by the specialist’s safety threshold $\theta_{D}$. Although the band of safety regulations where backfiring occurs can be narrow, our results suggest that this nonmonotonic effect of regulation exists for a broad set of games with different cost and revenue functions. Analytically, we prove that backfiring occurs for all quadratic-cost games in which the players invest any nonzero amount in both performance and safety without regulation.

## Second Insight: Properly Placed Safety Regulation Can Improve the Technology and the Players’ Utilities.

While our model shows weak regulation targeted predominantly at the domain-specialist can backfire, further analysis suggests that other regulatory regimes fare better. When safety standards are directed at both *G* and *D* with appropriate strength, regulation can improve not just safety, but the utilities of both players, defined as their revenue share minus their investment cost. This result might seem unintuitive: regulation only reduces the set of choices available to each actor in our model, so how can regulation lead to choices that mutually benefit both generalist and specialist? What is stopping the players from choosing utility-optimal strategies in the absence of regulation? The reason this phenomenon occurs is a Prisoner’s Dilemma-style result: the players’ unregulated strategies, which are chosen to maximize their individual utility, fail to yield the strategies that are globally optimal for both players. By constraining the actors away from the strategies that enable this kind of selfish behavior, regulation can act as a commitment device. The generalist can increase her investments in safety with the assurance that the domain specialist will contribute, too, rather than free-ride off of *G*’s efforts.

**Fig. 2.**
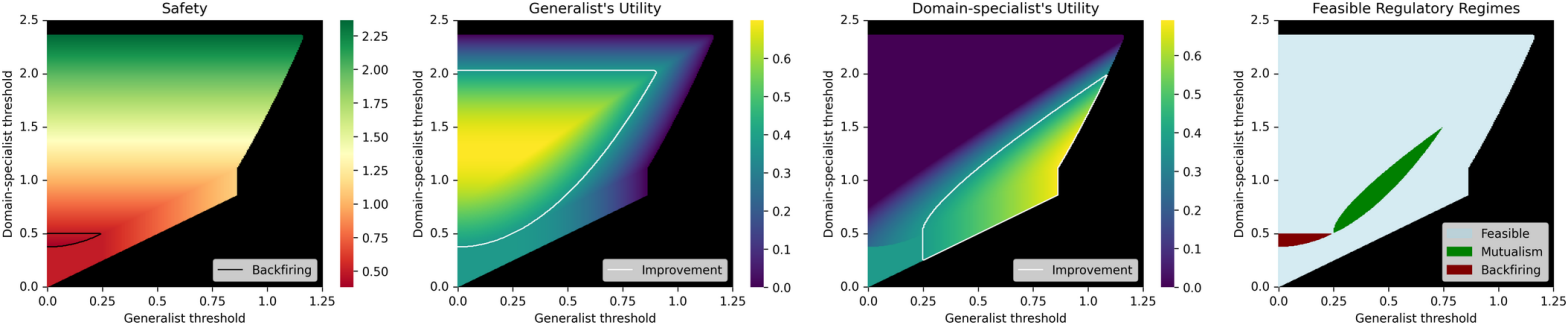
Simulated results for an example of the two-player AI regulation model with quadratic costs. Players make costly investments in performance and safety (visualized on left), and then receive some share of revenue that comes from the total investment levels. The players’ utilities—defined as their share of the revenue minus the cost of their investment—is visualized for the Generalist (second from left) and the Domain-Specialist (third from left). Colors represent different utility outcomes depending on different combinations of regulatory constraints $\left(\theta_{G},\theta_{D}\right)$ which constrain the players’ safety investments. The game is solved over a grid of plausible regulations: $\theta_{G}\in \left[0,1.25\right]$, $\theta_{D}\in \left[\theta_{G},2.5\right]$ using increments of 0.005, with a total of 105,651 simulated regulation games. Regulations that lead the players to abstain are depicted in black. There exists a region where nonzero regulation yields lower safety than no regulation (highlighted on leftmost plot). There also exists a region where regulation yields improvements to each players’ utility (highlighted on two center plots). The rightmost plot summarizes our results by showing the backfiring and mutualism outcomes in the $\theta_{G},\theta_{D}$ space. Parameter values for producing the plot: $C_{0}=C_{1}=I_{2}$, $r_{\alpha }=r_{\beta }=1$, and δ=0.5.

Games can exhibit both backfiring and mutualistic regulations, depending on who is targeted and at what threshold. For example, Fig. 2 depicts a particular instance of our game setting with one generalist and one domain-specialist. For the particular cost and revenue functions depicted, backfiring regulations and Pareto-improving regulations are possible, and the regulations yielding these effects are visualized. This figure represents a systematic sweep of all pairs of thresholds directed at the generalist, the domain specialist, or both. The pair of thresholds (0,0) corres- ponds to the case of no regulation. The safety implications of various regulations are depicted using a red-yellow-green color scale in the leftmost plot, while the utility implications for the generalist and specialist are depicted using a purple-green-yellow color scale in the center plots. Compared to the safety and utility values at the origin points, the backfiring and Pareto-improving regions are regulations which lead to lower safety and higher utilities for both players, respectively. Although this figure depicts an example of a single game, our analysis proves that these backfiring and Pareto-improving regulations exist for a broad class of games with quadratic costs. Namely, we find that backfiring occurs in all games in which the market incentivizes some nonzero investment in both performance and safety without regulation. Our characterization of when this phenomenon occurs includes separable scenarios (where the cost of investing in performance is independent of the cost of investing in safety), complementary scenarios (where investing in one makes the other cheaper), and weakly interfering scenarios (where the cost of investing in one makes the other more expensive) up to a certain bound, which we specify. We provide similar bounds for the mutualism results.

## Related Work

### AI Safety Regulation.

The rise of AI-related incidents have motivated several AI incident repositories to keep track of common risks (5, 6). Scholars have attempted to taxonomize AI harms to make sense of the growing array of incidents (7, 8). Some existing AI risk taxonomies organize risks primarily by domains, while others categorize these risks based on how they arise, including malicious use, malfunctions, or systemic effects from wide adoption (9). In our stylized model, we capture all such considerations using a single scalar that can be toggled by players through investments in safety. Common themes in policy drafts and recommendations stress the importance of balancing the goals of innovation and risk reduction, appropriately defining and targeting thresholds, and the impacts on incentives (10, 11).

### Game-Theoretic Models of AI Development.

A line of work uses formal models to reason about the strategic and social implications of machine learning (e.g., refs. 12–16). More recently, there have been proposals for using modeling approaches to understand the social and safety implications of generative AI (17, 18). Attempts to model the development process of generative AI often make use of the observation that development is sequential and involves multiple interacting actors (19). Many existing works explore different strategic aspects of the market for AI using a Stackelberg game. For example, Taitler et al. (20) use a sequential game to explore incentives for data-sharing. Further time-steps, players and decisions have been added to explore particular topics, including the level of openness and market entry dynamics (21, 22). Taitler et al. (23) introduce a particular notion of regulation in a related game-theoretic setting, and similar to our paper, they conceive of regulation as a restriction on the strategy space for developers of generative AI. Though work explicitly examining the interaction between performance and safety attributes in this setting is limited, Jagadeesan et al. (24) explores the interaction between these attributes in a linear regression setting in order to understand firms’ market entry decisions.

### The Fine-Tuning Games Model.

Our work builds on and extends the fine-tuning games model proposed by Laufer et al. (4). That model builds a one-dimensional game in which players must bargain over a revenue-sharing contract before investing in performance in sequence. We extend this model in two ways: first, the players’ strategy space is two-dimensional in our model, to capture the dynamic that often arises where a regulator wants to steer the technology in a direction (e.g., safety) other than that which is most-profitable (e.g., a baseline combination of performance and safety, dictated by the unregulated market). Second, we introduce the regulation, which can be seen as a floor constraining the feasible strategy space of each player. This allows us to explore when targeting generalists, specialists, both or neither is preferable for achieving desiderata like safety.

### Economic Theory and Contracts.

Our work leverages preexisting approaches that are common in the theoretical economics and game theory literature to reason about the set of possible impacts of AI safety regulation. In particular, we draw inspiration from canonical works in contract theory (25, 26) and the coordination of supply chains (27). Our model is a variant of a Principal-Agent problem in which the strategy space is defined by two real-valued attributes, and the cost and revenue are functions of these attributes. In this way, our model draws inspiration from Viscusi and Moore (28) analyzing the possible effects of product liability schemes on innovation and safety. That model—a one-player model with no order-of-play effects—demonstrates that liability does not, necessarily, hamper innovation. We assume that innovation is sequential, meaning that an entity’s investment in safety or performance builds on the contributions of past investments (29, 30).^‡^ In what we call the “no-regulation” game, we assume the players revenue-share via a linear contract (33), a common assumption in the literature (e.g., refs. 34–37). However, one way to interpret our mutualism results is as a demonstration that linear contracts are suboptimal in our setting. Our notion of regulation can be viewed as a set of nonlinear contracts defined by a set of strategy constraints, and our results suggest these more expressive contracts can yield higher utility. Still other forms of contracts are possible and may yield different utility implications. We leave these directions to future work.

## A Model of Regulating AI Safety

Here we offer a formal model for analyzing the effects of regulation on the development of AI applications. Our model is a sequence of subgames between two players. Each player will choose whether and how to contribute to the technology at a certain point in the development of the technology, and some revenue is received depending on the ultimate attributes of the technology. The players are constrained by regulatory floors on safety, which will be set exogenously by a regulator.

### Players.

A general-purpose producer, referred to as *G*, invests in a technology that may be adapted by domain-specialist(s), referred to as $D_{i}$. The generalist is the first to invest in the technology, meaning that before *G* moves, the technology’s attributes begin at value 0. Each specialist $D_{i}$ makes an investment after the generalist has moved.

### Technology.

We say a technology is described by one or more nonnegative attributes $\gamma \in R^{d}$. In this paper, we are interested in two attributes in particular: performance and safety.^§^ Unless otherwise specified, we assume d=2 and that $\gamma =\left[\alpha ,\beta \right]$ where α≥0 refers to performance and β≥0 refers to safety.

### Economic Interests.

Each player, acting in a way that maximizes their self-interest, invests some nonzero amount in the technology. *G* invests to $\gamma_{0}$ and each $D_{i}$ further invests to $\gamma_{i}$. Accordingly, each must pay a cost for their investment, $\phi_{0}\left(\gamma_{0}\right)$ and $\phi_{i}\left(\gamma_{i};\;\gamma_{0}\right)$, respectively. After both players invest, they share a revenue that is brought in as a function of the ultimate attributes of the technology in domain *i*, $r_{i}\left(\gamma_{i}\right)$. We assume that, for some $\delta_{i}\in \left[0,1\right]$, *G* gets $\delta_{i}r_{i}\left(\gamma_{i}\right)$ in revenue and $D_{i}$ gets $\left(1-\delta_{i}\right)r_{i}\left(\gamma_{i}\right)$. $\delta_{i}$ could either be exogenously fixed and given ahead of the game play, or it can be the result of bargaining between *G* and $D_{i}$. When we analyze a game with only one specialist, we will drop the subscript and use *δ*.

### Regulation.

We model regulation as imposed exogenously on the environment. Regulation is a minimum constraint on the safety investment that the players make. A regulation that targets *G*’s investment is characterized by a value $\theta_{G}\in R^{+}$. A nonzero regulation would constrain $G^{\prime }s$ strategy such that $\beta_{0}\geq \theta_{G}$. A regulation targeted at the domain-specialist, similarly, would take the form $\theta_{D}$ and lead the domain-specialist to be constrained in their strategy so $\beta_{i}\geq \theta_{D}$.

### Gameplay.

The game proceeds as a sequence of subgames:

- Regulation $\left\{\theta_{G},\theta_{D}\right\}$ is announced.
- *G* chooses to either abstain or invest in the technology, bringing it to
  $$\gamma_{0}=\left[\begin{array}{c} \alpha_{0}\\ \beta_{0} \end{array}\right].$$
- $D_{i}$ chooses to either abstain or invest in the technology, bringing it to
  $$\gamma_{i}=\left[\begin{array}{c} \alpha_{i}\\ \beta_{i} \end{array}\right],$$
  where $\alpha_{i}\geq \alpha_{0}$ and $\beta_{i}\geq \beta_{0}$.
- The technology brings in revenue $r_{i}\left(\gamma_{i}\right)$, which will be shared such that *G* receives $\delta_{i}r_{i}\left(\gamma_{i}\right)$ and *D* receives $\left(1-\delta_{i}\right)r_{i}\left(\gamma_{i}\right)$.

The utilities of the players are given below:

$$\begin{array}{cc} U_{G}:=\; & \sum_{i}\delta_{i}r_{i}\left(\gamma_{i}\right)-\phi_{0}\left(\gamma_{0}\right);\\ U_{D_{i}}:=\; & \left(1-\delta_{i}\right)r_{i}\left(\gamma_{i}\right)-\phi_{i}\left(\gamma_{i};\;\gamma_{0}\right). \end{array}$$

The best-response subgame perfect equilibrium strategy for the generalist and specialist, respectively, can be expressed as the following optimization problems:

$$\begin{array}{cc} \gamma_{0}^{*}:=\; & {\arg\max}_{\gamma_{0}}U_{G}\;s.t.\beta_{0}\geq \theta_{G};\\ \gamma_{i}^{*}:=\; & {\arg\max}_{\gamma_{i}}U_{D_{i}}\;s.t.\beta_{i}\geq \theta_{D}. \end{array}$$

Finally, the players will opt to abstain, if they prefer 0 utility to any other feasible strategy. If either player chooses to abstain, then both players receive 0 utility.

### Uses for the Conceptual Model.

Our model is game-theoretic in the sense that it sets up a system of interacting actors, each with decisions and incentives, and analyzes that system. We do not present empirical measurements of the world, but instead start from incentives and make inferences about the equilibrium strategies that arise in our model. Game theory papers historically operate at this level of generality for three reasons: First, it allows researchers to analyze and name emergent phenomena and strategies, contributing to theory. Second, when empirically observed instances are scant, it offers first principles for explaining or predicting aspects of the setting (though these principles can be challenged by subsequent analysis and observation). Third, it can inspire future empirical observations of instances where these arrangements of incentives exist in the world. Classic exemplars include the Prisoner’s Dilemma, Braess’s Paradox, and the Tragedy of the Commons—stylized incentive-driven mechanisms that subsequently informed both theory and measurement.

We employ this style and approach for reasoning about safety regulation along an AI development chain. Our model is sufficiently abstract that it should not guide decisions about certain features of regulation, including, for instance, whether to use taxation or product liability regimes as levers. Our model does not consider these features in order to provide a parsimonious conceptual model. Instead, our model offers a game-theoretic language for reasoning about the incentives at play, and reveals possibly counterintuitive phenomena that arise in a model. Accordingly, our claims should be interpreted as model-based existence results, rather than direct policy prescriptions. We welcome future work that might analyze new variants or alternative model settings, especially those tuned for particular policy interpretations, and discuss ideas for these directions in the Discussion section.

## Closed-Form Solutions

In this section, we analyze our regulation game where players’ cost functions can be expressed as a two-degree quadratic equation. Specifying a quadratic function over two attributes requires defining a matrix of cost coefficients. The cross-terms in this matrix represent how investments the attributes interact with one another. For the technical portions of the paper, we use the case of one domain specialist (*D*) as our focus. We therefore have the following cost and revenue functions:

$$\phi_{0}\left(\gamma_{0}\right)=\gamma_{0}^{T}C_{0}\gamma_{0},$$

$$\phi_{1}\left(\gamma_{1};\;\gamma_{0}\right)={\left(\gamma_{1}-\gamma_{0}\right)}^{T}C_{1}\left(\gamma_{1}-\gamma_{0}\right),$$

$$r\left(\gamma_{1}\right)=r^{T}\gamma_{1},$$

$$\begin{array}{c} \text{where}\;C_{0}=\left[\begin{array}{cc} c_{0,\alpha \alpha } & c_{0,\alpha \beta }\\ c_{0,\alpha \beta } & c_{0,\beta \beta } \end{array}\right],\;C_{1}=\left[\begin{array}{cc} c_{1,\alpha \alpha } & c_{1,\alpha \beta }\\ c_{1,\alpha \beta } & c_{1,\beta \beta } \end{array}\right],\\ \text{and}\;r=\left[\begin{array}{c} r_{\alpha }\\ r_{\beta } \end{array}\right]. \end{array}$$

The players’ utilities can thus be expressed as

$$U_{G}=\delta r^{T}\gamma_{1}-\gamma_{0}^{T}C_{0}\gamma_{0},$$

$$U_{D}=\left(1-\delta \right)r^{T}\gamma_{1}-{\left(\gamma_{1}-\gamma_{0}\right)}^{T}C_{1}\left(\gamma_{1}-\gamma_{0}\right).$$

It should be noted that not all values for the above parameters correspond to realistic or interesting scenarios. For example, we assume that the diagonal entries of both cost matrices $c_{0,\alpha \alpha },c_{0,\beta \beta },c_{1,\alpha \alpha },c_{1,\beta \beta }$ and the entries of the revenue vector $r_{\alpha },r_{\beta }$ are positive, to capture that investments in goods like safety and performance should have nonzero increasing cost and revenue. Although the cross-terms of the cost matrices can be negative, we require that $c_{0,\alpha \beta }>-\sqrt{c_{0,\alpha \alpha }c_{0,\beta \beta }}$ and $c_{1,\alpha \beta }>-\sqrt{c_{1,\alpha \alpha }c_{1,\beta \beta }}$, since it should not be that some combination of investments in α,β come at negative cost. Each players’ choices over *α* and *β* should be considered as simultaneous across the two attributes, representing a joint optimization over performance and safety.

In this section, we start by providing subgame perfect equilibria strategies in the case with no regulation, and then provide solutions for the regulated game. The form of problem we are dealing with is a continuous, not-necessarily-convex optimization problem with a constant number of constant-degree polynomials in a constant number of variables. Broadly, the strategy is to put forward a small number of candidate points that must be checked using a limited number of steps. These checks can be implemented numerically. After stating the solved subgame perfect equilibria strategies, we will move to a slate of numerical results and findings analyzing the effects of regulation.

### Subgame Perfect Equilibria Strategies Without Regulation.

In this section, we state the subgame perfect equilibrium strategies to the game under no regulation. These can provide intuition about the behaviors in the game, before we add the additional complexity of regulation. These can be seen as a strict generalization of the Fine-Tuning Games solutions (4) to games with two attributes that can interact.

Proposition 0.1. *Given an AI regulation game with quadratic costs, no regulation, and revenue-sharing parameter* *δ, domain specialist* *D’s subgame perfect equilibrium strategy is one of the values in the following set:*

$$\begin{array}{c} \gamma_{1}^{*}\in \left\{\begin{array}{c} \gamma_{0}+\frac{\left(1-\delta \right)}{2}C_{1}^{-1}r,\left[\begin{array}{c} \alpha_{0}\\ \beta_{0}+\frac{\left(1-\delta \right)r_{\beta }}{2c_{1\beta \beta }} \end{array}\right],\\ \left[\begin{array}{c} \alpha_{0}+\frac{\left(1-\delta \right)r_{\alpha }}{2c_{1\alpha \alpha }}\\ \beta_{0} \end{array}\right],\left[\begin{array}{c} \alpha_{0}\\ \beta_{0} \end{array}\right] \end{array}\right\} \end{array}$$

*The strategy is the feasible candidate which maximizes* $U_{D}$*,* *subject to* $U_{D}\geq 0,\alpha_{1}\geq \alpha_{0},\beta_{1}\geq \beta_{0}$.

Proposition 0.2. *Given a two-player AI regulation game with quadratic costs, no regulation, and revenue-sharing parameter* *δ,* *G’s best-response is one of the following candidates:*

$$\gamma_{0}^{*}\in \left\{\frac{\delta }{2}C_{0}^{-1}r,\left[\begin{array}{c} 0\\ \frac{\delta r_{\beta }}{2c_{0\beta \beta }} \end{array}\right],\left[\begin{array}{c} \frac{\delta r_{\alpha }}{2c_{0\alpha \alpha }}\\ 0 \end{array}\right],\left[\begin{array}{c} 0\\ 0 \end{array}\right]\right\}.$$

*The strategy is the candidate which maximizes* $U_{G}$*,* *subject to* $U_{G}\geq 0,U_{D}\geq 0,\alpha_{1}\geq 0,\beta_{1}\geq 0$.

The proofs of the above two propositions are given in *SI Appendix*. The solutions offer intuition about the set of strategies players might opt to take. They may venture in the direction of some combination of performance and safety, that is, move to a point that does not reside on either constraint. Or, alternatively, they may creep along the axes constraining their strategy space, and invest minimally in either performance or safety.

When do the players prefer one of these strategies over another? In general, our solutions are provided as sets of candidates because there are multiple intersecting constraints that must be checked to ensure a given candidate is optimal. However, our analysis reveals classes of games in which the market will lead players to invest in both safety and performance in conjunction under no regulation. We make this claim formal below.

Remark 0.3. Given the AI regulation game with quadratic costs, no regulation, and revenue-sharing parameter $\delta \in \left(0,1\right)$. If any player *p*’s cost interaction term satisfies the following inequalities:

$$c_{p,\alpha \beta }<\;\min\left(\sqrt{c_{p,\alpha \alpha }c_{p,\beta \beta }},\frac{c_{p,\alpha \alpha }r_{\beta }}{r_{\alpha }},\frac{c_{p,\beta \beta }r_{\alpha }}{r_{\beta }}\right),$$

then their best-response strategy includes nonzero investment in both performance and safety.

This claim is proven in *SI Appendix*. The broad intuition is that the first of these inequalities establishes the costs are strictly convex, and the second two ensure that the player’s cost interactions are not so positive that investing in both performance and safety is prohibitively expensive compared to investing in one or the other alone. The claim offers some intuition for when a player prefers to invest in both attributes together, even without regulation pushing them to invest in safety. It covers all games in which the cost interactions are negative, which we call the complementary scenario, meaning it is cheaper to invest in both performance and safety together than to invest in each individually. It further covers all games in which the cost interactions are zero, which we call the separable scenario, meaning there is no benefit or loss to investing in both attributes in conjunction. Finally, it covers certain instances where the cost interactions are positive, which we call the interfering scenario, meaning safety investments make performance more costly, and vice versa.

### Subgame Perfect Equilibria Strategies with Regulation.

Here we provide the subgame perfect equilibria strategies of the two players in our two-attribute game, in the presence of regulation. Notice that the no-regulation gameplay can be derived from these solutions simply by plugging in $\theta_{D}=\theta_{G}=0$. Like the solutions in the prior section, these generalized solutions require checking a number of candidates, but this number has grown to account for the possible responses to regulation.

Proposition 0.4. *Given a two-attribute fine-tuning game with quadratic costs, regulatory constraints* $\theta_{G},\theta_{D}$*,* *and bargaining parameter* *δ,* *the domain specialist* *D’s* *subgame perfect equilibrium strategy is one of the values in the following set:*

$$\gamma_{1}^{*}\in \left\{\begin{array}{c} \gamma_{0}+\frac{\left(1-\delta \right)}{2}C_{1}^{-1}r,\left[\begin{array}{c} \alpha_{0}\\ \beta_{0}+\frac{\left(1-\delta \right)r_{\beta }}{2c_{1\beta \beta }} \end{array}\right],\\ \left[\begin{array}{c} \alpha_{0}+\frac{\left(1-\delta \right)r_{\alpha }}{2c_{1\alpha \alpha }}-\frac{c_{1\alpha \beta }}{c_{1\alpha \alpha }}\max\left(0,\theta_{D}-\beta_{0}\right)\\ \max(\beta_{0},\theta_{D}) \end{array}\right],\\ \left[\begin{array}{c} \alpha_{0}\\ \max(\beta_{0},\theta_{D}) \end{array}\right],abstain \end{array}\right\}$$

*The strategy is the feasible candidate which maximizes* $U_{D}$*,* *subject to* $U_{D}\geq 0,\alpha_{1}\geq \alpha_{0},\beta_{1}\geq \;\max\left(\beta_{0},\theta_{D}\right)$.

Proposition 0.5 *Given a two-attribute, two-player fine-tuning game with quadratic costs, regulatory constraints* $\theta_{G},\theta_{D}$*,* *and bargaining parameter* *δ,* *G’s* *best-response is one of the following candidates:*

- $\frac{\delta }{2}C_{0}^{-1}r,$
- $\left[\begin{array}{c} 0\\ \frac{\delta r_{\beta }}{2c_{0\beta \beta }} \end{array}\right],$
- $\left[\begin{array}{c} \frac{\delta r_{\alpha }}{2c_{0\alpha \alpha }}-\frac{c_{0\alpha \beta }}{c_{0\alpha \alpha }}\theta_{G}\\ \theta_{G} \end{array}\right],$
- $\left[\begin{array}{c} 0\\ \theta_{G} \end{array}\right],$
- abstain,
- *Three additional candidates along the* $U_{D}=0$ *constraint, which is given by the following quadratic equation:*
  [1] $$\begin{array}{cc} 0= & \left(1-\delta \right)r_{\alpha }\alpha_{0}+\frac{{\left(1-\delta \right)}^{2}r_{\alpha }^{2}}{4c_{1\alpha \alpha }}+\left(1-\delta \right)r_{\beta }\theta_{D}\\ & -\frac{c_{1\alpha \beta }}{c_{1\alpha \alpha }}\left(1-\delta \right)r_{\alpha }\theta_{D}+\frac{c_{1\alpha \beta }^{2}}{c_{1\alpha \alpha }}\theta_{D}^{2}-c_{1\beta \beta }\theta_{D}^{2}\\ & +\left(\frac{c_{1\alpha \beta }}{c_{1\alpha \alpha }}\left(1-\delta \right)r_{\alpha }-2\frac{c_{1\alpha \beta }^{2}}{c_{1\alpha \alpha }}\theta_{D}+2c_{1\beta \beta }\theta_{D}\right)\beta_{0}\\ & +\left(\frac{c_{1\alpha \beta }^{2}}{c_{1\alpha \alpha }}-c_{1\beta \beta }\right)\beta_{0}^{2}. \end{array}$$

*The strategy is the candidate which maximizes* $U_{G}$, subject to $U_{G}\geq 0,U_{D}\geq 0,\alpha_{1}\geq 0,\beta_{1}\geq \theta_{G}$.

The proof of the above propositions is provided in *SI Appendix*. We outline the intuition behind the proof as follows: notice that the optimization is an inequality-constrained quadratic optimization problem. The problem has been set up so no solutions exist at infinity, that is, the solutions will either be local maxima or will reside on constraints. Therefore, we can find the critical points for the unconstrained problem, as well as the critical points for every possible combination of every constraint in our problem. This yields a set of candidates, which are worked out and listed in the set above.

There is a bit of additional subtlety in the process for arriving at the last three candidates along the constraint listed at the end of the Proposition. Two of the three candidates reside at the intersection of this constraint with the other constraints—that is, they satisfy the constraint listed and either $\alpha_{0}=0$ or $\beta_{0}=\theta_{G}$. Finding the point that satisfies these combinations of constraints is only as hard as solving the roots of a one-variable quadratic, at worst. The third one, however, is a bit more convoluted. This candidate can be described as the solution to the optimization problem $\max_{\gamma_{0}}U_{G}\;\text{subject to}\;U_{D}=0$, where the other constraints are ignored. Although this is a (not necessarily convex) quadratic program, specifying the Lagrangian suggests that its solution must be the solution of a system of three distinct equations with three unknown variables $\left(\alpha_{0},\beta_{0},\lambda \right)\in R^{3}$. Two of these equations are quadratic, and the other is linear:

- $\delta r_{\alpha }-2c_{0\alpha \alpha }\alpha_{0}-2c_{0\alpha \beta }\beta_{0}-\lambda \left(1-\delta \right)r_{\alpha }=0$,
- $\frac{\delta c_{1\alpha \beta }r_{\alpha }}{c_{1\alpha \alpha }}-2c_{0\beta \beta }\beta_{0}\;-2c_{0\alpha \beta }\alpha_{0}\;-\lambda \left(\frac{c_{1\alpha \beta }}{c_{1\alpha \alpha }}\left(1-\delta \right)r_{\alpha }-2\frac{c_{1\alpha \beta }^{2}\theta_{D}}{c_{1\alpha \alpha }}+2\left(\frac{c_{1\alpha \beta }}{c_{1\alpha \alpha }}-c_{1\beta \beta }\right)\beta_{0}\right)=0$,
- The quadratic stated in the proposition.

Though there may be multiple roots satisfying the above equations, the roots are bounded in typical fashion by Bezout’s Theorem. Further algebra for arriving at solutions is left to the computer.

## Computational Results

Here we describe a set of numerical tests and demonstrations to explore the strategies in our game, using the solved strategies from the previous section. Our analysis here is focused on the existence of a persistent facet of the model concerning the way the players shift their strategies in response to regulation. With the knowledge that one player or the other is required to meet a regulatory floor, agents can choose their strategies accordingly. In a variety of cases, we observe that the strategies shift in a way that lowers the ultimate safety investment compared to safety attained under no regulation. This effect—which we term backfiring—is observable in cases where the regulation is weak, meaning it imposes a floor that the players already meet under no regulation.

This section starts by demonstrating the existence of this effect. We then discuss its persistence in cases where players can flexibly choose how they share revenue via a linear contract. Finally, in stark contrast to the observation that regulation can backfire, we find that regulation can act as a commitment device, unlocking strategy sequences that mutually benefit the players.

### Regulation Can Backfire.

**Fig. 3.**
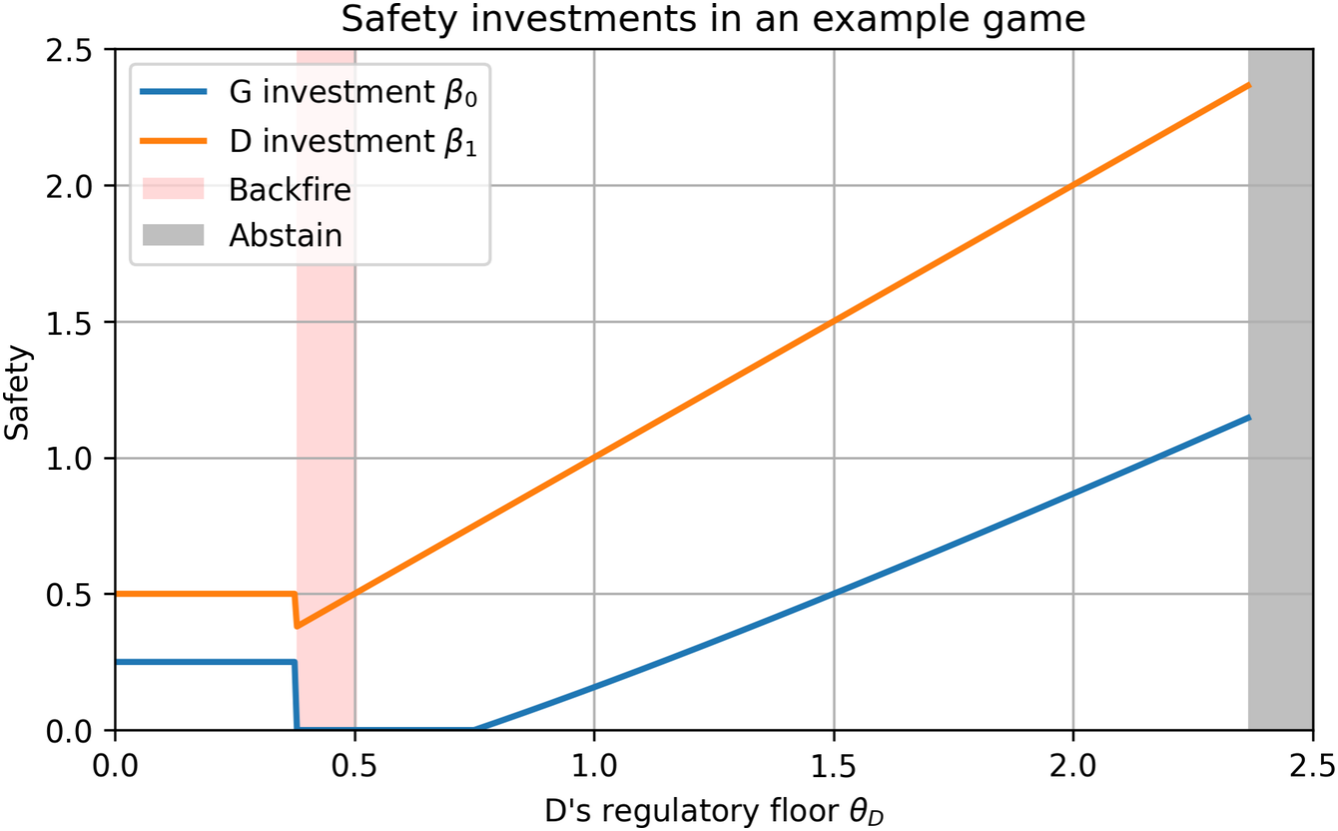
Backfiring observed in a basic two-player game where $\theta_{G}=0$ and $\theta_{D}$ is varied over the range [0,2.5]. As $\theta_{D}$ is swept upward from 0, there is some value at which the generalist’s score exhibits a discontinuity and the investment in safety lowers. In this example, the discontinuity occurs at a threshold value below the safety attained under no regulation (0.5). In response to this discontinuity in the generalist’s strategy, the domain-specialist minimally complies with the regulation, meaning the ultimate safety is reduced for some nonzero regulations. The $\beta_{1}$ curve conveys information redundant with the first panel of Fig. 2, where the regulation is swept only along the vertical line in which the generalist’s threshold is 0.

Consider a basic game given by the following set of cost and revenue parameters: $C_{1}=C_{0}=I_{2},r_{\alpha }=r_{\beta }=1,\delta =0.5$. This game is separable, meaning there are no interaction effects between performance and safety, and it assumes the market without regulation places equal value on performance and safety. Fig. 3 depicts the players’ strategies in this game, for varying levels of regulation targeting the Domain-specialist alone. For the lowest regulatory thresholds, we observe that the players stick to their no-regulation safety investments, since they already clear the threshold and their no-regulation investments remain optimal. As the regulatory floor is increased, however, the generalist’s strategy exhibits a discontinuity. Crucially, this drop in G’s safety investment occurs at a regulatory threshold lower than the no-regulation safety strategy.

Why does *G* switch strategies? Here we attempt to provide some intuition. Absent regulation, there exists some typical best-response that *D* will take, and *G* must anticipate this best-response to choose an optimal strategy. Even if *G* would theoretically prefer *D* to invest more in safety, *G* cannot fully control *D*’s actions. Regulation that targets *D*, however, does just this: it restricts *D*’s strategy space so *D* must commit to certain safety investments, regardless of *G*’s strategy. Therefore, in the presence of regulation, *G* is incentivized to select a new strategy sequence with lower investments in safety, because *G* knows that *D* will cover the gap in safety between *G*’s investment and *D*’s threshold. Put another way, *G* is given the opportunity to engage in a kind of free-riding behavior. *G* creates a gap in the safety investment as a cost-cutting exercise, knowing *D* must bridge the gap to reap any reward in the game.

Our results here convey that backfiring occurs in one instance of the game. We have yet to give a clear characterization of how widespread this phenomenon is. How wide is the region of regulatory thresholds that cause backfiring, and what do we know about the magnitude of safety reductions? From our analysis so far, we can intuit that backfiring only occurs when the regulation is weak, meaning the safety thresholds targeted at each player is lower than the no-regulation safety investment. We can also observe that the mechanism leading to backfiring is enabled when the generalist can minimize their safety investment as a cost-cutting strategy, which suggests the band of backfiring regulations should be widest when only the domain-specialist is targeted. In order to further characterize this effect, we note that the example we have shown so far assumes the revenue-sharing parameter is fixed at δ=0.5. One might imagine that, instead of a fixed revenue sharing arrangement, players can collectively decide how to share the revenue. Does the ability to influence the revenue-sharing parameter prevent cases where weak regulation backfires? We turn to this question next.

### Bargaining Does Not Prevent Backfiring.

**Fig. 4.**
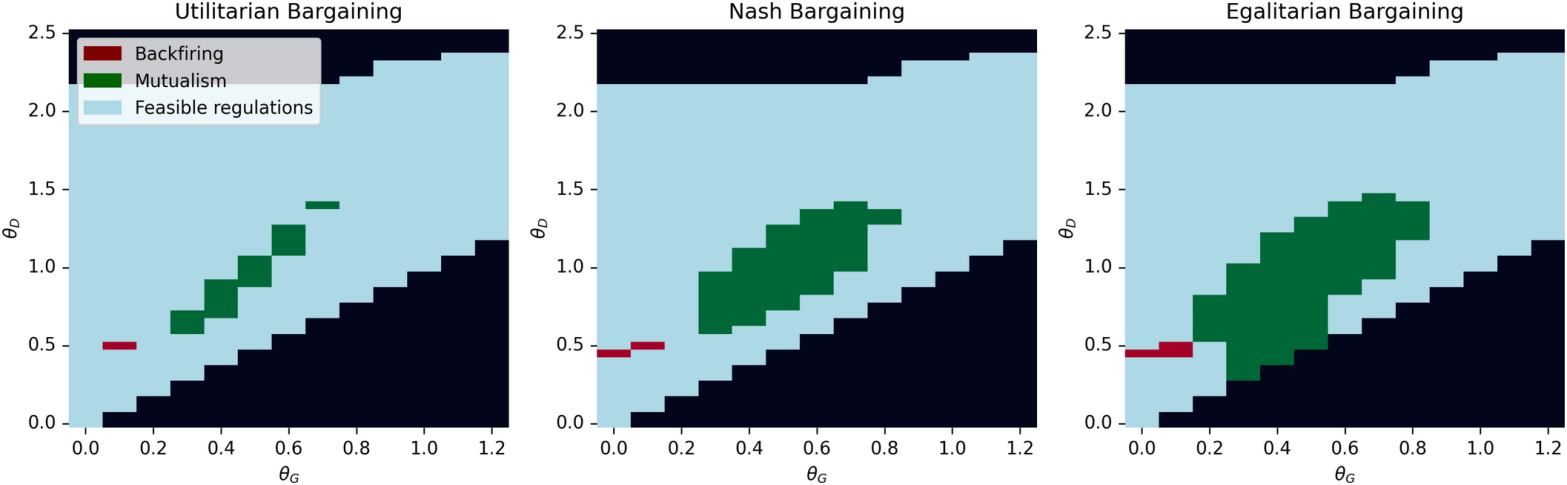
Results from numerical tests over the set of possible $\left(\theta_{G},\theta_{D}\right)$ pairs in the two-attribute, two-player, separable quadratic-cost game. Backfiring occurs in the weak regulatory regimes in which $\theta_{D}$ is just below $\beta_{0}^{A}$. Regulations that mutually improve both players’ utilities overanarchy are detected for all three bargaining solutions. The highest aggregate utility in this game is achieved at $\theta_{G}=0.5,\theta_{D}=1$. Here the observed backfiring scenarios are constricted to a narrow set of parameter values with modest safety impacts whereas the mutualism scenarios occur for a wider set of regulations.

Here we provide evidence that the existence of backfiring persists in more games beyond the examples portrayed in Fig. 2. In particular, we relax the assumption that players share their revenue according to a constant revenue-sharing parameter δ=0.5. Instead, we allow players to reach bargaining agreements to distribute revenue—and, correspondingly, profit—in a way that maximizes their joint utility. Bargaining solutions are arrangements that maximize the players’ joint utility.^¶^ We provide evidence that even when players can distribute revenue in a way that maximizes the joint utility, these arrangements can still exhibit backfiring effects. We assume here that the players jointly agree on a bargaining solution before either invests effort, but after learning about the regulation.^#^Fig. 4 shows the numerical results for a variant of the separable game where we vary the value of *δ* over 98 values in the range [0.01,0.99]. We vary the regulatory setting for 13 $\theta_{G}$ values in [0,1.2] and 51 $\theta_{D}$ values in [0,2.5], for a total of 49,686 simulated games. The figure depicts three different processes for arriving at an optimal bargain: utilitarian, which selects *δ* to maximize the sum of utilities, Nash, which selects *δ* to maximize the product of utilities (38), and egalitarian, which sets *δ* to maximize the minimum of the utilities. In all scenarios, we observe at least one instance of a combination of regulations that backfire. Further, we observe a cluster of regulation regimes that yield mutual improvement to utility. The results suggest that even in instances where players can choose how to distribute revenue through revenue-sharing, these agreements are suboptimal for engendering the right sort of commitment from each of the players, if their goal is to mutually benefit from their interaction. In the next section, we explore the idea that regulation can bring about a mutualistic benefit, beyond what is achievable through bargaining.

### Regulation Can Act as a Commitment Device.

**Fig. 5.**
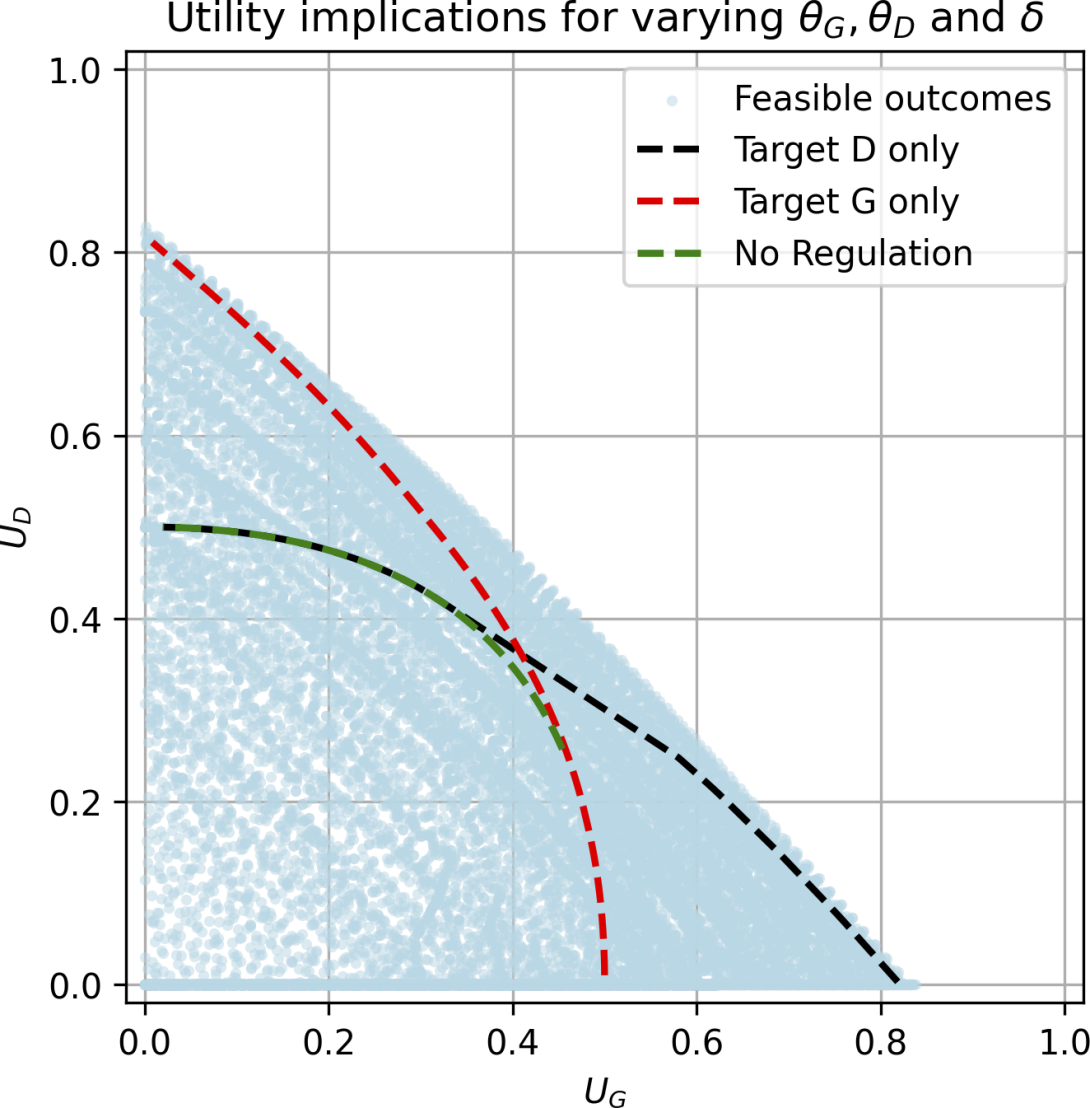
The set of attainable utility outcomes over a grid of possible regulation regimes and bargains for the two-attribute, two-player, separable quadratic-cost game. If we imagine that regulations are endogenous to the game—that is, regulations are decided collectively by the players, like *δ*, then each of the blue points represents a possible game with utility implications for the two players. If the players are restricted to a particular regulatory regime—targeting G only, D only, or neither—then the utility they are able to achieve (depicted in dashed lines) suffers, compared to the regime where both players are subjected to regulation. The shape formed by the light blue dots represents the full set of simulated games, suggesting that regulation targeting both players is at times needed to achieve outcomes that cannot be achieved by regulating just one.

Here we show that there exist cases where regulation can leave both players better off than anarchy, while also benefiting the safety of the technology. Even though the regulation constrains the space of investments that players are able to achieve, it can nonetheless leave each player with higher utility than they are able to achieve under no regulation. To make this finding more clear, we depict the set of all achievable $\left(U_{G},U_{D}\right)$ combinations in Fig. 5. The light blue cloud of points represents all attainable utility scenarios, over a grid of $\theta_{D},\theta_{G},$ and *δ* values. The dotted lines represent the convex hull (northeastern faces) of attainable utility implications for the following regimes: 1) neither player is targeted with regulation (depicted in green), 2) one player is targeted with regulation (depicted in red and black), and 3) both players are targeted with regulation (inferrable from the outermost feasible points). The figure suggests that a nonvacuous constraint on both players achieves more preferable utility outcomes than regulations of individual players or bargaining alone are able to achieve.

These results suggest that, although regulation can backfire, it can also mutually serve the interests of both players while also improving the level of safety of the technology. This finding raises the following question: if it was possible to achieve higher utilities all around, why was this set of strategies not chosen by the players in the unregulated game? The players did not opt for this set of strategies because these strategies are dominated for at least one player in at least one subgame. As a hypothetical, imagine that under no regulation, the players sit down for a conversation before the game, and both say they will contribute *ϵ* additional investment in safety. When the game reaches the last step, however, *D* finds he benefits more from investing only $\frac{\left(1-\delta \right)}{2}C_{1}^{-1}$, rather than the agreed upon value of $\frac{\left(1-\delta \right)}{2}C_{1}^{-1}+2\epsilon$. What’s more, *G* knows that *D* will do this, and so *G*’s decision will break the agreement before *D* even gets the chance to respond. Without the regulation restricting *D*’s behavior away from changing strategies in the final hour, nothing prevents *D* from pursuing the highest-utility strategy, even if it harms *G*. Thus, our model has a prisoner’s dilemma dynamic baked into it: there are feasible strategies that leave both players better off, but these strategies are not equilibria.

Absent regulation, the players might wish they could ensure the other will uphold their side of a verbal agreement, though they are unable to guarantee it. Regulation, therefore, can act as a commitment device, which lends teeth to agreements that the players are able to enter prior to making their investments. This commitment device can be valuable in a formal sense: both players would be willing to pay for it, as long as the price is less than the amount of utility they collectively gain under regulation.

## A General Characterization

In the previous sections, we arrived at closed-form solutions for the players’ strategies and have demonstrated individual instances that exhibit the backfiring effect of regulation. We have not yet determined how widespread this phenomenon is. In this section, we provide analytical results that characterize when this phenomenon occurs. Our findings suggest that this effect is notably widespread. We find that for all quadratic-cost games, backfiring occurs as long as both of the technology’s attributes (performance and safety) are sufficiently complementary such that, under no regulation, the players will invest in some combination of them. Intuitively, if the players invested only in performance under no regulation, backfiring would be impossible as the baseline safety investment would be zero. Therefore, our condition for backfiring covers all games where the market prefers some nonzero baseline investment in performance and safety. The condition we rely on is precisely the condition introduced in *Remark* 0.3, which represents an upper bound on the cost interaction terms. This section will prove that both backfiring and mutualism occur in a range of scenarios that depend crucially on the cost interaction term, and will describe what this dependence looks like.

### Backfiring Occurs in All Mixed-Strategy Games.

Below we prove that for all AI regulation games in which the players invest a nonzero amount in safety and performance under no regulation, there is a nonempty set of regulatory regimes that exhibit a backfiring effect.

Theorem 0.6 *Given an AI regulation game with quadratic costs. If both players’ cost interactions meet the following conditions:*

$$c_{p,\alpha \beta }<\;\min\left(\sqrt{c_{p,\alpha \alpha }c_{p,\beta \beta }},\frac{c_{p,\alpha \alpha }r_{\beta }}{r_{\alpha }},\frac{c_{p,\beta \beta }r_{\alpha }}{r_{\beta }}\right),$$

*then there exists an* ϵ>0 *such that the regulatory regime* $\theta_{G}=0,\theta_{D}=\beta_{0}^{A}-\epsilon$ *backfires.*

The proof of the above theorem is provided in *SI Appendix*. Here we provide an overview of the conceptual argument. We start by observing that the unregulated optimal strategies $\gamma_{0}^{A},\gamma_{1}^{A}$ remain feasible in weak regulatory settings. These strategies dominate all alternative strategies in which the players contribute to safety beyond their regulatory constraints, as any such strategy was available in the no regulation scenario, so they were already shown to be suboptimal compared to $\gamma_{0}^{A},\gamma_{1}^{A}$. The proof’s task, therefore, is to find some $\theta_{D}<\beta_{1}^{A}$ and some $\gamma_{0}^{\prime }\neq \gamma_{0}^{A}$, such that *D* minimally complies with the regulation ($\beta_{0}^{\prime }=\theta_{D})$, and further, $U_{G}\left(\gamma_{0}^{\prime };\;\theta_{D}\right)>U_{G}\left(\gamma_{0}^{A};\;\theta_{D}\right)$. For the proof to work, we choose a regulation of $\theta_{G}=0,\theta_{D}=\beta_{0}^{A}-\epsilon$ for some small positive ϵ>0, and generalist strategy $\gamma_{0}^{\prime }=\left[\begin{array}{c} \frac{\delta r_{\alpha }}{2c_{0,\alpha \alpha }}\left(\beta_{0}^{A}-2\epsilon \right)\\ \beta_{0}^{A}-2\epsilon \end{array}\right]$. For sufficiently small *ϵ*, we find that the change to the utility of *G* for using this strategy is positive as long as the following condition is met: $r_{\beta }>\frac{c_{1,\alpha \beta }}{c_{1,\alpha \alpha }}r_{\alpha }$. This inequality, given by the analysis in *SI Appendix*, is precisely the condition established in Remark 0.3 for nonzero investment in safety under no regulation.

The above results demonstrate that backfiring does not only exist in rare degenerate cases: it occurs in a range of scenarios in which players share revenue and each contribute nonzero effort to the development of the technology. These scenarios include settings in which the two attributes are complementary, as well as a range of settings where the two attributes are interfering, up to a particular limit that we are able to specify. We note that further generalizations are open for broader functional forms, including more expressive polynomial costs and exponential costs. The generality of the backfiring effect in the quadratic model gives us reason to believe that the effect might hold for a broader set of forms, though we leave these directions to future work.

### Mutualism Occurs in Sufficiently Separable Games.

So far, we have shown that a set of regulations backfire in a swath of two-attribute games. Here we provide a second result on a set of regulations that fare better. Using similar logic about games with bounded interaction effects between the two attributes, we find that there exist combinations of regulatory thresholds that mutually improve the two players’ utilities, as well as the safety level of the technology. We state this result below.

Theorem 0.7 *Given a two-player AI regulation game with quadratic costs. If both players meet the following conditions:*

$$|c_{p,\alpha \beta }|<\;\min\left(\sqrt{c_{p,\alpha \alpha }c_{p,\beta \beta }},\frac{c_{p,\alpha \alpha }r_{\beta }}{r_{\alpha }},\frac{c_{p,\beta \beta }r_{\alpha }}{r_{\beta }}\right),$$

*then there exists an* ϵ>0 *such that the regulatory regime* $\theta_{G}=\beta_{0}^{A}+\epsilon ,\theta_{D}=\beta_{1}^{A}+2\epsilon$ *mutually improves both players’ utilities.*

The proof of the above theorem is given in *SI Appendix*. The proof follows a similar strategy to the backfiring proof. We are focused on the set of games where the players arrive at unconstrained solutions in the case of no regulation, and we perturb the regulation by a small positive *ϵ* value and see the implications for the players’ utilities. Here, instead of targeting only the domain-specialist and specifying a threshold slightly below the unconstrained optimal strategy, we set the regulation to target both players using a threshold slightly above their unconstrained strategies. Instead of measuring the impact on safety, we measure the impact on the players’ utilities and find that, under the specified condition, the utilities both improve.

The results suggest that, similar to the characterization of backfiring, the mutualism effect is observable in a range of quadratic-cost games, including in separable scenarios and a range of complementary and interfering scenarios. Notice, however, that our condition for establishing when mutualism occurs is slightly different than the condition in the backfiring theorem. Instead of a one-sided bound on the players’ cost interaction terms, our proof relies on a two-sided bound. The analysis suggests there may be certain games where the two attributes are strongly complementary where slightly increasing the regulation in the manner proposed does not increase players’ utilities. In other words, if the market already sufficiently incentivizes joint investments in safety and performance, then forcing safety requirements on both players in equal proportion may not benefit players’ utilities. In these cases, a linear contract may suffice to serve the utilities of the players, and so regulation would only be needed for advancing safety; it would not serve the additional role of enforcing commitments from players for their mutual benefit.

## Discussion

Proposals for AI regulation have made use of the idea that different entities contribute to these technologies in succession. This work provides a stylized model for reasoning about the effects of targeting AI safety regulation along this development chain. Through our analysis of this model, we find that *weak* safety regulation predominantly targeted at the domain specialist can backfire, yielding lower investments in safety than in the alternative case of no regulation. We further find that regulation appropriately targeted at both upstream producers and downstream specialists can exhibit a mutualism effect in which both entities benefit. After demonstrating instances of the backfiring and mutualism effects through a numerical simulation, we provide an analysis showing these phenomena are not just degenerate cases but hold in a range of parameterized scenarios.

### Comparison to Existing Cases.

There are few observable examples of the dynamics and effects of AI safety regulation at the time of this paper’s submission, which in part motivates our theoretical approach to reasoning about the incentive effects of policies. Still, we can look to past examples of regulations of other (non-AI) technologies to offer examples of how incentive effects play out and can be significant considerations for forward-looking regulations.

Consider the legal landscape in 1995–96 that led to Section 230, a famous piece of U.S. regulation that exempts internet service providers from the publishing-related liabilities faced by major news sources and print publications (39). At the time, two major providers—CompuServe and Prodigy—took markedly different approaches to moderation, in that CompuServe did not moderate at all, whereas Prodigy hired a team of content moderators. The eventual appearance of harmful (e.g., libelous or defamatory) content on both platforms led to a pair of court cases in which Prodigy was found liable for their content because they moderated (40), whereas CompuServe was not liable because they did not moderate (41). By passing Section 230, Congress acted in part because of the worry that this pair of cases could incentivize internet service providers to avoid moderating, while the low-resource and often anonymous posters would similarly avoid accountability because few would go after them. This, in certain ways, reflects the dynamics in our model because it is a case where a limited amount of regulatory enforcement led to incentive effects that did not induce the desired behavior. There are further similarities because the regulatory regime of holding the downstream posters, editors, or moderators liable could have possibly encouraged a “free-riding” strategy where the provider is incentivized to avoid any moderation so as to not accept liability. There are no doubt important differences between the AI regulation setting and the context surrounding Section 230. For example, the latter concerns liability for speech and expression, which triggers a different set of statutes and stipulations from regulations around safety (in part due to the existence of the First Amendment in the United States, which is specific to the context of speech). However, we provide this case as we believe it offers one example where a very weak or nonexistent regulatory regime was preferred to a weak regulatory regime.

For another comparison, consider regulation of large technology platforms’ labor strategies. Many online marketplace firms rely on contracted work for driving and delivery. In these arrangements, individuals or third-party logistics (3PL) partners perform tasks for pay, and disputes over the workers’ status (either as employees or as contractor) determine who bears respon- sibility for wages, benefits, workplace protections, and liability. This setting resembles our model because downstream providers offer services to consumers, but to do so, they rely on an upstream internet platform. Ballot measures including California’s Proposition 22 (42) establish modest minimum benefits for workers—including a wage guarantee during rides and a conditional stipend toward health expenses—while classifying drivers not as employees but as independent contractors. Even though these provisions create minimum protections for workers, one study estimated that hourly earnings can fall well below the state’s minimum wage when idle time and expenses are accounted for (43).^‖^ Still, platforms spent record amounts on the campaign to pass Proposition 22 (44) and worked to pass similar measures in Massachusetts (45) and elsewhere, suggesting these regulations advance the interests of platforms.

These dynamics around contract labor arise not just over worker benefits but also over safety. When incidents harm workers or members of the public, platforms might point to workers’ contractor status to limit their own liability. For exam- ple, a series of investigations documented fatal crashes involving firms contracted by Amazon to make deliveries, along with strict financial and managerial policies Amazon instituted that placed pressure on contractors (46, 47). Still, Amazon denies liability in such incidents, arguing that the contractor is responsible. This is perhaps an example of a setting in which a general-purpose provider might be incentivized to undercut the investments in safety because they have the ability to direct regulatory requirements at third parties, which just barely or nominally comply. To avoid these dynamics, labor regulators have advocated for the concept of “joint employment,” which suggests that labor protection duties are shared between the multiple entities involved in designing the workers’ contract terms (48). This approach seemingly mirrors our analysis of cases in which both entities are targeted with regulatory requirements in some proportion.

This worker protection setting is distinct from the AI regulation setting because there already exists a backdrop of many laws and policies that regulate employment in various jurisdictions, including minimum wage laws. Though no comparison is perfect, we provide these examples to motivate the importance of incentive modeling and game theory in technology regulation.

### Future Directions.

Our results reveal natural directions for future research. In the setting we have put forward, it would be interesting to move beyond showing the existence of backfiring and mutualism regions and characterize the shape of these regions. Certain segments of the boundaries of these regions are straightforward but others seem to require solving higher-order polynomials to express in closed-form.

Generalizations beyond the quadratic-cost games might be interesting. For instance, it may be possible to show that backfiring and Pareto-improvement effects occur for any convex cost and concave revenue games meeting some conditions on the functions’ characteristics, e.g., their slopes and intercepts.

There are several features of the AI ecosystem that fall outside our simple setup but could materially affect incentives, offering novel lines of future research. For example, we have predominantly focused on the case where there is one domain-specialist, but in many real-world settings the development of AI technologies involve multiple domains, and each domain may contain many entities who compete. To what extent does competition between multiple entities change the backfiring and Pareto-improving impacts of regulation? Pursuing questions about multiple domain-specialists would require further specifying the structure of *G*’s contract with each specialist, which might reasonably be conceived as a constant revenue share across domains, a constant fixed price across domains, or a variable price across domains. Relatedly, approaches to regulating different specialists may be conceived of as domain-specific (different requirements for each domain) or domain-agnostic (requirements for all domains). Domain-specific policies have the advantage of configuring safety requirements depending on domain, which might allow for more robust approaches to regulation, though they may require more bespoke regulatory efforts. The advantages and disadvantages of domain-specific regulatory efforts might be weighed against the possibilities of backfiring and free-riding effects.

Pursuing questions about multiple generalists may also illuminate interesting directions. These approaches might draw from literature on firms with multiple suppliers (49). Healthy competition between base model providers could plausibly mitigate backfiring effects, because specialists would be incentivized to contract with providers who have invested sufficiently in safety. A model with multiple generalist providers could further exhibit divide-and-conquer strategies: If different domains have different preferences over attributes, there may be scenarios where general providers specialize their investments to capture some domains and cede others to their competitors. Further analysis could explore the conditions under which a particular generalist might prefer to vertically integrate with a domain specialist, via an umbrella firm with a specified bargaining and revenue-sharing mechanisms.

The state of global regulation can inspire further variants of the model where there exist more than one regulator. The emergence of AI companies and their market strategies depend on the regulatory environment, and some companies might even selectively choose to be headquartered in a given jurisdiction because they are drawn by favorable regulation. The fragmented state of regulation could inspire variants of the model where there exists some degree of competition between regulators, and firms exert some choice over which jurisdiction they operate in. These and other directions raise questions about how to design regulation to account for rich constellations of interacting actors.

## Supplementary Material

Appendix 01 (PDF)

## Data Availability

There are no data underlying this work. Code for producing the numerical results and creating the figures is available on GitHub (https://github.com/bendlaufer/ai-regulation-games) (50).

## Footnotes

^*^Such AI models are at times referred to as “foundation” or “frontier” models (1, 2). Throughout this paper, we will use the terminology of general-purpose AI to refer to large-scale models that can be adapted to a range of tasks and domains.

^†^There may be scenarios where the regulation is too high, or the generalist’s investment is too low, such that the domain specialist gives up entirely and abstains from any involvement in the technology’s production. We handle these scenarios in our analysis of the model, but our point here is to demonstrate that there are circumstances in which regulation gives the generalist leverage to free-ride by lowering the initial investment in safety.

^‡^However, some have observed that safety investments can *degrade* as the result of fine-tuning performance investments especially when model weights are open (31, 32). This scenario, and especially the interaction effects with model openness, are ripe areas for further analysis.

^§^The results and inferences we draw may hold for other attributes that relate to one another with similar structure.

^¶^The relevance of bargaining solutions to our setting is described in further depth by Laufer et al. (4).

^#^The next sections will relax this assumption further, providing findings on the existence of backfiring and mutualism for every nontrivial linear revenue-sharing agreement δ∈(0,1).

^‖^We note that there exists a deluge of studies and conflicting views on the topic.
