# Supplementary material for: The backfiring effect of weak AI safety regulation
Source: Proc Natl Acad Sci U S A. 2026 Jul 20;123(30):e2509768123. doi: 10.1073/pnas.2509768123 (PMC13416633; doi:10.1073/pnas.2509768123)
Supplement: Supplementary file 1 — Appendix 01 (PDF) [file pnas.2509768123.sapp.pdf]

PNAS

Supporting Information for

The Backfiring Effect of Weak AI Safety Regulation

Benjamin Laufer, Jon Kleinberg and Hoda Heidari

Corresponding Author Jon Kleinberg.
E-mail: kleinberg@cornell.edu

This PDF file includes:

Supporting text

Supporting Information Text

Game Solving

A. Player's strategies without regulation. **The domain-specialist's strategy.** The proof for Proposition 4.1 is given below.

Proposition 4.1. *Given an AI regulation game with quadratic costs, no regulation, and revenue-sharing parameter δ, domain specialist D's subgame perfect equilibrium strategy is one of the values in the following set:*

$$\gamma_1^* \in \left\{ \gamma_0 + \frac{(1-\delta)}{2} C_1^{-1} r, \begin{bmatrix} \alpha_0 \\ \beta_0 + \frac{(1-\delta) r_\beta}{2 c_{1\beta\beta}} \end{bmatrix}, \begin{bmatrix} \alpha_0 + \frac{(1-\delta) r_\alpha}{2 c_{1\alpha\alpha}} \\ \beta_0 \end{bmatrix}, \begin{bmatrix} \alpha_0 \\ \beta_0 \end{bmatrix} \right\}$$

The strategy is the feasible candidate which maximizes U_D, subject to $U_D \geq 0, \alpha_1 \geq \alpha_0, \beta_1 \geq \beta_0$.

Proof. D's best-response strategy is the value γ_1^* that maximizes D's utility.

$$\gamma_1^*(\gamma_0, \delta) = \arg\max_{\gamma_1} U_D(\gamma_0, \gamma_1 \delta) \text{ s.t. } U_D \geq 0, \alpha_1 \geq \alpha_0, \beta_1 \geq \beta_0$$

Observe that D will not abstain because zero-investment ($\gamma_1 = \gamma_0$) is cost-free, yielding non-negative utility, so we can safely ignore the constraint. To solve the optimization, we specify the Lagrangian as follows for some multipliers $\lambda_1 \in \mathbb{R}, \lambda_2 \in \mathbb{R}$ and a slack variables $s_1 \in \mathbb{R}, s_2 \in \mathbb{R}$. By construction, we assert that the slack variables are only non-zero when the multipliers are zero, and the multipliers are non-zero only if the slack variables are zero.

$$\mathcal{L} := (1-\delta) r^T \gamma_1 - (\gamma_1 - \gamma_0)^T C_1 (\gamma_1 - \gamma_0) - \lambda_1(\alpha_1 - \alpha_0 - s_1^2) - \lambda_2(\alpha_1 - \alpha_0 - s_2^2).$$

We partially differentiate with respect to each decision variable and each multiplier.

$$\begin{aligned}
\frac{\partial}{\partial \alpha_1} \mathcal{L} &= 0 \\
\iff (1-\delta) r_\alpha - 2c_{1,\alpha\alpha}(\alpha_1 - \alpha_0) + 2c_{1,\alpha\beta}(\beta_1 - \beta_0) - \lambda_1 &= 0 \\
\frac{\partial}{\partial \beta_1} \mathcal{L} &= 0 \\
\iff (1-\delta) r_\beta - 2c_{1,\beta\beta}(\beta_1 - \beta_0) + 2c_{1,\alpha\beta}(\alpha_1 - \alpha_0) - \lambda_2 &= 0 \\
\frac{\partial}{\partial \lambda_1} \mathcal{L} &= 0 \\
\iff -\alpha_1 + \alpha_0 + s_1^2 &= 0 \\
\frac{\partial}{\partial \lambda_2} \mathcal{L} &= 0 \\
\iff -\beta_1 + \beta_0 + s_2^2 &= 0
\end{aligned}$$

Using complementary slackness, we have four possible options:

1. $s_1 = 0, \lambda_1 > 0, s_2 = 0, \lambda_2 > 0 \rightarrow \beta_1^* = \beta_0, \alpha_1^* = \alpha_0$.
2. $s_1 \neq 0, \lambda_1 = 0, s_2 = 0, \lambda_2 > 0 \rightarrow \beta_1^* = \beta_0$, and we can plug into our first of four equations above:
$$\begin{aligned}
(1-\delta) r_\alpha - 2c_{1,\alpha\alpha}(\alpha_1 - \alpha_0) + 2c_{1,\alpha\beta}(\beta_1 - \beta_0) - \lambda_1 &= 0 \\
\rightarrow (1-\delta) r_\alpha - 2c_{1,\alpha\alpha}(\alpha_1 - \alpha_0) &= 0 \\
\rightarrow \alpha_1^* = \alpha_0 + \frac{(1-\delta) r_\alpha}{2c_{1,\alpha\alpha}}.
\end{aligned}$$
3. $s_1 = 0, \lambda_1 > 0, s_2 \neq 0, \lambda_2 = 0 \rightarrow \alpha_1^* = \alpha_0$, and we can plug in to equation 2:
$$\begin{aligned}
(1-\delta) r_\beta - 2c_{1,\beta\beta}(\beta_1 - \beta_0) + 2c_{1,\alpha\beta}(\alpha_1 - \alpha_0) - \lambda_2 &= 0 \\
\rightarrow (1-\delta) r_\beta - 2c_{1,\beta\beta}(\beta_1 - \beta_0) - \lambda_2 &= 0 \\
\rightarrow \beta_1^* = \beta_0 + \frac{(1-\delta) r_\beta}{2c_{1,\beta\beta}}.
\end{aligned}$$
4. $s_1 \neq 0, \lambda_1 = 0, s_2 \neq 0, \lambda_2 = 0 \rightarrow$ This is the unconstrained critical point, and is solved via the first two systems of equations:
$$\begin{aligned}
\nabla U_D = (1-\delta) r - 2C_1(\gamma_1 - \gamma_0) &= 0 \\
\rightarrow \gamma_1^* = \gamma_0 + \frac{(1-\delta)}{2} C_1^{-1} r.
\end{aligned}$$

Thus we have established our four candidates in the proposition statement. □

The Generalist's strategy. The proof for Proposition 4.2 is given below.

Proposition 4.2. *Given a two-player AI regulation game with quadratic costs, no regulation, and revenue-sharing parameter δ, G's best-response is one of the following candidates:*

$$\gamma_0^* \in \left\{ \frac{\delta}{2} C_0^{-1} r, \begin{bmatrix} 0 \\ \frac{\delta r_\beta}{2c_{0\beta\beta}} \end{bmatrix}, \begin{bmatrix} \frac{\delta r_\alpha}{2c_{0\alpha\alpha}} \\ 0 \end{bmatrix}, \begin{bmatrix} 0 \\ 0 \end{bmatrix} \right\}$$

The strategy is the candidate which maximizes U_G, subject to $U_G \geq 0, U_D \geq 0, \alpha_1 \geq 0, \beta_1 \geq 0$.

Proof. G's best-response strategy is the value γ_0^* that maximizes G's utility.

$$\gamma_0^*(\delta) \quad = \quad \arg\max_{\gamma_0} U_G(\gamma_0, \delta) \text{ s.t. } U_G \geq 0, \alpha_0 \geq 0, \beta_0 \geq 0.$$

Following the same steps as the proof of Proposition 4.1, we specify the Lagrangian as follows for multipliers $\lambda_1 \in \mathbb{R}, \lambda_2 \in \mathbb{R}$ and a slack variables $s_1 \in \mathbb{R}, s_2 \in \mathbb{R}$.

$$\mathcal{L} := \delta r^T \gamma_1 - \gamma_0^T C_0 \gamma_0 - \lambda_1(\alpha_0 - s_1^2) - \lambda_2(\alpha_0 - s_2^2).$$

We partially differentiate with respect to each decision variable and each multiplier.

$$\begin{aligned} \frac{\partial}{\partial \alpha_0}\mathcal{L} &= 0 \\ \iff \delta r_\alpha - 2c_{0,\alpha\alpha}\alpha_0 + 2c_{0,\alpha\beta}\beta_0 - \lambda_1 &= 0, \\ \frac{\partial}{\partial \beta_1}\mathcal{L} &= 0 \\ \iff \delta r_\beta - 2c_{0,\beta\beta}\beta_0 + 2c_{1,\alpha\beta}\alpha_0 - \lambda_2 &= 0, \\ \frac{\partial}{\partial \lambda_1}\mathcal{L} &= 0 \\ \iff -\alpha_0 + s_1^2 &= 0, \\ \frac{\partial}{\partial \lambda_2}\mathcal{L} &= 0 \\ \iff -\beta_0 + s_2^2 &= 0. \end{aligned}$$

Using complementary slackness, we have four possible options:

1. $s_1 = 0, \lambda_1 > 0, s_2 = 0, \lambda_2 > 0 \to \beta_0^* = 0, \alpha_0^* = 0.$
2. $s_1 \neq 0, \lambda_1 = 0, s_2 = 0, \lambda_2 > 0 \to \beta_0^* = 0$, and we can plug into our first of four equations above:

$$\begin{aligned} \delta r_\alpha - 2c_{0,\alpha\alpha}\alpha_0 + 2c_{0,\alpha\beta}\beta_0 - \lambda_1 &= 0 \\ \to \delta r_\alpha - 2c_{0,\alpha\alpha}\alpha_0 &= 0 \\ \to \alpha_0^* = \frac{\delta r_\alpha}{2c_{0,\alpha\alpha}}. \end{aligned}$$

3. $s_1 = 0, \lambda_1 > 0, s_2 \neq 0, \lambda_2 = 0 \to \alpha_0^* = 0$, and we can plug in to equation 2:

$$\begin{aligned} \delta r_\beta - 2c_{0,\beta\beta}\beta_0 + 2c_{0,\alpha\beta}\alpha_0 - \lambda_2 &= 0 \\ \delta r_\beta - 2c_{0,\beta\beta}\beta_0 &= 0 \\ \to \beta_0^* = \frac{\delta r_\beta}{2c_{0,\beta\beta}}. \end{aligned}$$

4. $s_1 \neq 0, \lambda_1 = 0, s_2 \neq 0, \lambda_2 = 0 \to$ This is the unconstrained critical point, and is solved via the first two systems of equations:

$$\begin{aligned} \nabla U_G = \delta r - 2C_0\gamma_0 &= 0 \\ \to \gamma_0^* = \frac{\delta}{2} C_0^{-1} r. \end{aligned}$$

Thus we have established our four candidates. □

B. Condition for non-zero performance and safety investment. Condition establishing non-zero investment. Below we prove Remark 4.3.

Remark 4.3. *Given the AI regulation game with quadratic costs, no regulation, and revenue-sharing parameter $\delta \in (0,1)$. If any player p's cost interaction term satisfies the following inequalities:*

$$c_{p,\alpha\beta} < \min\left(\sqrt{c_{p,\alpha\alpha}c_{p,\beta\beta}}, \frac{c_{p,\alpha\alpha}r_\beta}{r_\alpha}, \frac{c_{p,\beta\beta}r_\alpha}{r_\beta}\right),$$

then their best-response strategy includes non-zero investment in both performance and safety.

Proof. The first of the three inequalities establishes that the player's costs are strictly convex:

$$c_{\alpha\beta} < \sqrt{c_{p,\alpha\alpha}c_{p,\beta\beta}} \iff c_{p,\alpha\alpha}c_{p,\beta\beta} - c_{\alpha\beta}^2 > 0 \iff \det C_p > 0.$$

By the spectral theorem, we know a 2x2 matrix is positive definite if and only if its determinant and trace are both positive, which is now established. By Lemma 1, the utility is strictly concave for our setting if and only if the cost is strictly convex. Thus the unconstrained solution is the global optimum as long as it is feasible. Thus, the necessary and sufficient condition for optimality is the condition for feasibility.

- For the generalist:

$$\frac{\delta}{2}C_0^{-1}r > 0 \iff \frac{\delta}{2\det C_0}\begin{bmatrix} c_{0,\beta\beta}r_\alpha - c_{0,\alpha\beta}r_\beta \\ -c_{0,\alpha\beta}r_\alpha + c_{0,\alpha\alpha}r_\beta \end{bmatrix} > \begin{bmatrix} 0 \\ 0 \end{bmatrix}$$

  Using the same positive definiteness identity above, we know the determinant is positive. We are given $\delta > 0$. Thus we can cancel the positive constant term $\frac{\delta}{2\det C_0}$. The two inequalities simplify to those stated in the proposition.

- For the specialist, the proof proceeds identically. Observe that $(1-\delta) \geq 0$ and the unconstrained contribution is given by: $\frac{1-\delta}{2}C_1^{-1}r$.

□

C. Proof for Player Strategies with Regulation. Here we provide proofs for our propositions establishing best-response strategies for the players.

Domain-specialist best-response under regulation. Here we provide the proof of Proposition 4.4, the domain specialist's best response under regulatory requirement θ_D.

Proposition 4.4. *Given a two-attribute fine-tuning game with quadratic costs, regulatory constraints θ_G, θ_D, and bargaining parameter δ, the domain specialist D's subgame perfect equilibrium strategy is one of the values in the following set:*

$$\gamma_1^* \in \left\{ \begin{array}{c} \gamma_0 + \frac{(1-\delta)}{2}C_1^{-1}r, \begin{bmatrix} \alpha_0 \\ \beta_0 + \frac{(1-\delta)r_\beta}{2c_{1\beta\beta}} \end{bmatrix}, \begin{bmatrix} \alpha_0 + \frac{(1-\delta)r_\alpha}{2c_{1\alpha\alpha}} - \frac{c_{1\alpha\beta}}{c_{1\alpha\alpha}}\max(0, \theta_D - \beta_0) \\ \max(\beta_0, \theta_D) \end{bmatrix}, \\ \begin{bmatrix} \alpha_0 \\ \max(\beta_0, \theta_D) \end{bmatrix}, \texttt{abstain}. \end{array} \right\}$$

The strategy is the feasible candidate which maximizes U_D, subject to $U_D \geq 0, \alpha_1 \geq \alpha_0, \beta_1 \geq \max(\beta_0, \theta_D)$.

Proof. D's best-response strategy is the value γ_1^* that maximizes D's utility. D will abstain if and only if the best option yields negative utility.

$$\gamma_1^*(\gamma_0, \delta, \theta_D) \quad = \quad \arg\max_{\gamma_1} U_D(\gamma_0, \delta, \theta_D) \text{ s.t. } U_D \geq 0, \alpha_1 \geq \alpha_0, \beta_1 \geq \max(\beta_0, \theta_D).$$

Define $\kappa = \max(\beta_0, \theta_D)$. To solve the optimization, we specify the Lagrangian as follows for some multipliers $\lambda \in \mathbb{R}^3$ and a slack variables $s \in \mathbb{R}^3$.

$$\mathcal{L} := (1-\delta)r^T\gamma_1 - (\gamma_1 - \gamma_0)^T C_1(\gamma_1 - \gamma_0) - \lambda_1(\alpha_1 - \alpha_0 - s_1^2) - \lambda_2(\beta_1 - \kappa - s_2^2) - \lambda_3(U_D - s_3^2).$$

We partially differentiate with respect to each decision variable and each multiplier.

$$
\begin{aligned}
\frac{\partial}{\partial \alpha_1}\mathcal{L} &= 0\\
\iff (1-\delta)r_\alpha - 2c_{1,\alpha\alpha}(\alpha_1-\alpha_0) + 2c_{1,\alpha\beta}(\beta_1-\kappa) - \lambda_1 - \lambda_3\frac{\partial U_D}{\partial \alpha_1} &= 0\\
\iff (1-\lambda_3)\left((1-\delta)r_\alpha - 2c_{1,\alpha\alpha}(\alpha_1-\alpha_0) + 2c_{1,\alpha\beta}(\beta_1-\kappa)\right) - \lambda_1 &= 0\\
\frac{\partial}{\partial \beta_1}\mathcal{L} &= 0\\
\iff (1-\delta)r_\beta - 2c_{1,\beta\beta}(\beta_1-\kappa) + 2c_{1,\alpha\beta}(\alpha_1-\alpha_0) - \lambda_2 - \lambda_3\frac{\partial U_D}{\partial \beta_1} &= 0\\
\iff (1-\lambda_3)\left((1-\delta)r_\beta - 2c_{1,\beta\beta}(\beta_1-\kappa) + 2c_{1,\alpha\beta}(\alpha_1-\alpha_0)\right) - \lambda_2 &= 0\\
\frac{\partial}{\partial \lambda_1}\mathcal{L} &= 0\\
\iff -\alpha_1+\alpha_0+s_1^2 &= 0\\
\frac{\partial}{\partial \lambda_2}\mathcal{L} &= 0\\
\iff -\beta_1+\kappa+s_2^2 &= 0\\
\frac{\partial}{\partial \lambda_3}\mathcal{L} &= 0\\
\iff -U_D+s_2^2 &= 0\\
\iff -(1-\delta)r^T\gamma_1 + (\gamma_1-\gamma_0)^T C_1(\gamma_1-\gamma_0) + s_2^2 &= 0
\end{aligned}
$$

Using complementary slackness, we have eight possible options:

1. $s_1 = 0, \lambda_1 > 0, s_2 = 0, \lambda_2 > 0, s_3 \neq 0, \lambda_3 = 0 \to \beta_1^* = \kappa, \alpha_1^* = \alpha_0$.

2. $s_1 = 0, \lambda_1 > 0, s_2 = 0, \lambda_2 > 0, s_3 = 0, \lambda_3 > 0 \to \beta_1^* = \kappa, \alpha_1^* = \alpha_0$. This offers the same candidate as (1).

3. $s_1 = 0, \lambda_1 > 0, s_2 \neq 0, \lambda_2 = 0, s_3 \neq 0, \lambda_3 = 0 \to \alpha_1^* = \alpha_0$, solve equations (1) and (2) for β_1^* and λ_1. Omitting the algebra, this yields:
$$\gamma_1^* = \begin{bmatrix} \alpha_0 \\ \beta_0 + \frac{(1-\delta)r_\beta}{2c_{1\beta\beta}} \end{bmatrix}$$

4. $s_1 = 0, \lambda_1 > 0, s_2 \neq 0, \lambda_2 = 0, s_3 = 0, \lambda_3 > 0 \to \alpha_1^* = \alpha_0$, solve equations (1) and (2) for β_1^* and λ_1. This solution, if it is distinct from the previous solution (3), will always be dominated because it is characterized by 0 utility for G.

5. $s_1 \neq 0, \lambda_1 = 0, s_2 = 0, \lambda_2 > 0, s_3 = 0, \lambda_3 > 0 \to \beta_1^* = \kappa \to$ solve equations (1) and (2) for λ_1 and α_1^*. Omitting algebra, this yields:
$$\gamma_1^* = \begin{bmatrix} \alpha_0 + \frac{(1-\delta)r_\alpha}{2c_{1\alpha\alpha}} - \frac{c_{1\alpha\beta}}{c_{1\alpha\alpha}}\max(0, \theta_D - \beta_0) \\ \max(\beta_0, \theta_D) \end{bmatrix}$$

6. $s_1 \neq 0, \lambda_1 = 0, s_2 = 0, \lambda_2 > 0, s_3 \neq 0, \lambda_3 = 0 \to$ this solution, if it is distinct from the previous one (5), will always be dominated because it is characterized by 0 utility for G.

7. $s_1 \neq 0, \lambda_1 = 0, s_2 \neq 0, \lambda_2 = 0, s_3 \neq 0, \lambda_3 = 0 \to \alpha_1^* = \alpha_0$, solve equations (1) and (2) for α_1^*, β_1^*. This is the unconstrained solution. Omitting algebra, this yields: $\gamma_1^* = \gamma_0 + \frac{(1-\delta)}{2}C_1^{-1}r$.

8. $s_1 \neq 0, \lambda_1 = 0, s_2 \neq 0, \lambda_2 = 0, s_3 = 0, \lambda_3 > 0 \to \beta_1^* = \kappa \to$ solve equations (1) and (2) for α_1^*, β_1^*. This solution, if it is distinct from (7), will always be dominated by (7) because it is characterized by 0 utility for G.

Thus we have established our four candidates in the proposition statement. To handle the `abstain` scenario, we check each candidate produced in the process above by plugging the strategy to our formula for U_D. If none yield positive utility, then the domain specialist prefers to `abstain`. □

The generalist's subgame perfect equilibrium strategy under regulation. Here we prove Proposition 4.5.

Proposition 4.5. *Given a two-attribute, two-player fine-tuning game with quadratic costs, regulatory constraints θ_G, θ_D, and bargaining parameter δ, G's best-response is one of the following candidates:*

- $\frac{\delta}{2}C_0^{-1}r$,

- $\begin{bmatrix} 0 \\ \frac{\delta r_\beta}{2c_{0\beta\beta}} \end{bmatrix}$,
- $\begin{bmatrix} \frac{\delta r_\alpha}{2c_{0\alpha\alpha}} - \frac{c_{0\alpha\beta}}{c_{0\alpha\alpha}}\theta_G \\ \theta_G \end{bmatrix}$,
- $\begin{bmatrix} 0 \\ \theta_G \end{bmatrix}$,
- *abstain*,
- *Three additional candidates along the $U_D = 0$ constraint, which is given by the following quadratic equation:*

$$(1-\delta)r_\alpha\alpha_0 + \left(\frac{(1-\delta)^2 r_\alpha^2}{4c_{1\alpha\alpha}} + (1-\delta)r_\beta\theta_D - \frac{c_{1\alpha\beta}}{c_{1\alpha\alpha}}(1-\delta)(r_\alpha)\theta_D + \frac{c_{1\alpha\beta}^2}{c_{1\alpha\alpha}}\theta_D^2 - c_{1\beta\beta}\theta_D^2\right) +$$
$$\left(\frac{c_{1\alpha\beta}}{c_{1\alpha\alpha}}(1-\delta)r_\alpha - 2\frac{c_{1\alpha\beta}^2}{c_{1\alpha\alpha}}\theta_D + 2c_{1\beta\beta}\theta_D\right)\beta_0 + \left(\frac{c_{1\alpha\beta}^2}{c_{1\alpha\alpha}} - c_{1\beta\beta}\right)\beta_0^2 = 0.$$

The strategy is the candidate which maximizes U_G, subject to $U_G \geq 0, U_D \geq 0, \alpha_1 \geq 0, \beta_1 \geq \theta_G$.

Proof. G's best-response strategy is the value γ_0^* that maximizes G's utility.

$$\gamma_0^*(\delta, \theta_G, \theta_D) \quad = \quad \arg\max_{\gamma_0} U_G(\gamma_0; \delta, \theta_G, \theta_D) \text{ s.t. } U_G \geq 0, U_D \geq 0, \alpha_0 \geq 0, \beta_0 \geq \theta_G.$$

To solve the optimization, we specify the Lagrangian as follows for some multipliers $\lambda \in \mathbb{R}^4$ and a slack variables $s \in \mathbb{R}^4$.

$$\mathcal{L} := \quad \delta r^T\gamma_1 - \gamma_0^T C_0\gamma_0 - \lambda_1(\alpha_0 - s_1^2) - \lambda_2(\beta_1 - \theta_G - s_2^2) - \lambda_3(U_D - s_3^2) - \lambda_4(U_G - s_4^2).$$

We partially differentiate with respect to each decision variable and each multiplier.

$$\begin{aligned}
\frac{\partial}{\partial\alpha_1}\mathcal{L} &= 0 \\
\iff \delta r_\alpha - 2c_{0,\alpha\alpha}\alpha_0 + 2c_{0,\alpha\beta}\beta_0 - \lambda_1 - \lambda_3\frac{\partial U_G}{\partial\alpha_0} - \lambda_4\frac{\partial U_D}{\partial\alpha_0} &= 0 \\
\iff (1-\lambda_3)\left(\delta r_\alpha - 2c_{0,\alpha\alpha}\alpha_0 + 2c_{0,\alpha\beta}\beta_0\right) - \lambda_1 & \\
-\lambda_4\left((1-\delta)r_\alpha - 2c_{1,\alpha\alpha}(\alpha_1-\alpha_0) + 2c_{1,\alpha\beta}(\beta_1 - \max(\beta_0,\theta_D))\right) &= 0, \\
\frac{\partial}{\partial\beta_1}\mathcal{L} &= 0 \\
\iff \delta r_\beta - 2c_{0,\beta\beta}\beta_0 + 2c_{0,\alpha\beta}\alpha_0 - \lambda_2 - \lambda_3\frac{\partial U_G}{\partial\beta_0} - \lambda_4\frac{\partial U_D}{\partial\beta_0} &= 0 \\
\iff (1-\lambda_3)\left(\delta r_\beta - 2c_{0,\beta\beta}\beta_0 + 2c_{0,\alpha\beta}\alpha_0\right) - \lambda_2 & \\
-\lambda_4\left((1-\delta)r_\beta - 2c_{1,\beta\beta}(\beta_1 - \max(\beta_0,\theta_D) + 2c_{1,\alpha\beta}(\alpha_1-\alpha_0)\right) &= 0, \\
\frac{\partial}{\partial\lambda_1}\mathcal{L} &= 0 \\
\iff -\alpha_0 + s_1^2 &= 0, \\
\frac{\partial}{\partial\lambda_2}\mathcal{L} &= 0 \\
\iff -\beta_0 + s_2^2 &= 0, \\
\frac{\partial}{\partial\lambda_3}\mathcal{L} &= 0 \\
\iff -U_G + s_2^2 &= 0 \\
\iff -\delta r^T\gamma_1 + \gamma_0^T C_1\gamma_0 + s_2^2 &= 0, \\
\frac{\partial}{\partial\lambda_3}\mathcal{L} &= 0 \\
\iff -U_D + s_2^2 &= 0 \\
\iff -(1-\delta)r^T\gamma_1 + (\gamma_1-\gamma_0)^T C_1(\gamma_1-\gamma_0) + s_2^2 &= 0.
\end{aligned}$$

Using complementary slackness, we have sixteen possible options. For brevity, we refer to these options by the constraints they satisfy, where **bold** corresponds to the constraints being activated. The algebra is omitted for exposition; only the candidates yielded are noted for each constraint setting.

1. $\boldsymbol{\alpha_0}, \boldsymbol{\beta_0}, \boldsymbol{U_G}, \boldsymbol{U_D} \to [0, \theta_G]$.
2. $\boldsymbol{\alpha_0}, \boldsymbol{\beta_0}, \boldsymbol{U_G}, U_D \to [0, \theta_G]$
3. $\boldsymbol{\alpha_0}, \boldsymbol{\beta_0}, U_G, U_D \to [0, \theta_G]$
4. $\boldsymbol{\alpha_0}, \boldsymbol{\beta_0}, U_G, \boldsymbol{U_D} \to [0, \theta_G]$
5. $\boldsymbol{\alpha_0}, \beta_0, \boldsymbol{U_G}, \boldsymbol{U_D} \to \begin{bmatrix} 0 \\ \frac{\delta r_\beta}{2c_{0\beta\beta}} \end{bmatrix}$
6. $\boldsymbol{\alpha_0}, \beta_0, \boldsymbol{U_G}, U_D \to \begin{bmatrix} 0 \\ \frac{\delta r_\beta}{2c_{0\beta\beta}} \end{bmatrix}$
7. $\boldsymbol{\alpha_0}, \beta_0, U_G, U_D \to \begin{bmatrix} 0 \\ \frac{\delta r_\beta}{2c_{0\beta\beta}} \end{bmatrix}$
8. $\boldsymbol{\alpha_0}, \beta_0, U_G, \boldsymbol{U_D} \to$ One of three along $U_D = 0$ curve.
9. $\alpha_0, \boldsymbol{\beta_0}, \boldsymbol{U_G}, \boldsymbol{U_D} \to \gamma_0^* = \begin{bmatrix} \frac{\delta r_\alpha}{2c_{0\alpha\alpha}} - \frac{c_{0\alpha\beta}}{c_{0\alpha\alpha}}\theta_G \\ \theta_G \end{bmatrix}$.
10. $\alpha_0, \boldsymbol{\beta_0}, \boldsymbol{U_G}, U_D \to \begin{bmatrix} \frac{\delta r_\alpha}{2c_{0\alpha\alpha}} - \frac{c_{0\alpha\beta}}{c_{0\alpha\alpha}}\theta_G \\ \theta_G \end{bmatrix}$
11. $\alpha_0, \boldsymbol{\beta_0}, U_G, U_D \to \begin{bmatrix} \frac{\delta r_\alpha}{2c_{0\alpha\alpha}} - \frac{c_{0\alpha\beta}}{c_{0\alpha\alpha}}\theta_G \\ \theta_G \end{bmatrix}$
12. $\alpha_0, \boldsymbol{\beta_0}, U_G, \boldsymbol{U_D} \to$ Two of three along the $U_D = 0$ curve.
13. $\alpha_0, \beta_0, \boldsymbol{U_G}, \boldsymbol{U_D} \to \frac{\delta}{2} C_0^{-1} r$.
14. $\alpha_0, \beta_0, \boldsymbol{U_G}, U_D \to \frac{\delta}{2} C_0^{-1} r$
15. $\alpha_0, \beta_0, U_G, U_D \to \frac{\delta}{2} C_0^{-1} r$
16. $\alpha_0, \beta_0, U_G, \boldsymbol{U_D} \to$ Three of three along the $U_D = 0$ curve.

Thus we have established our four candidates in the proposition statement. To handle the `abstain` scenario, we check each candidate produced in the process above by plugging the strategy to our formula for U_G. If none yield positive utility, then the generalist prefers to `abstain`. □

1. Helper Lemmas and Analysis

Here we write out helper Lemmas and analysis for our proofs concerning backfiring and mutualism.

Lemma 1. *In the AI regulation game with quadratic costs, any player's utility is strictly concave if and only if their cost matrix is positive definite.*

Proof. The generalist utility function is given by $U_G = \delta r^T \gamma_1 - \gamma_0^T C_0 \gamma_0$. Observe this is twice differentiable everywhere. Thus the function is strictly concave in α_0, β_0 if and only if its Hessian derivative is negative definite. We compute the Hessian as follows:

$$H := \begin{bmatrix} \frac{\partial^2 U_G}{\partial \alpha_0^2} & \frac{\partial^2 U_G}{\partial \alpha_0 \partial \beta_0} \\ \frac{\partial^2 U_G}{\partial \beta_0 \partial \alpha_0} & \frac{\partial^2 U_G}{\partial \beta_0^2} \end{bmatrix} = -2C_0.$$

This matrix is negative definite if and only if C_0 is positive definite.

The proof for the domain specialist follows the same steps. □

Lemma 2. *In any AI regulation game with separable quadratic costs, if there is no regulation, both players will invest a non-zero amount in each attribute.*

Proof. By Lemma 1, we are given that the utilities are strictly concave. Thus, the proof consists of showing that 1) the utility function is greater than or equal to 0 at the origin point of zero investment and 2) the gradient points towards the interior of the feasible set everywhere along the boundaries.

Here we prove the two conditions for U_G:

1. $U_G(\alpha_0 = 0, \beta_0 = 0) = \delta r^T \vec{0} - 0 = 0$

2. We prove this for each constraint, $\alpha_0 \geq 0, \beta_0 \geq 0$:

- $\frac{\partial U_G}{\partial \alpha_0}\Big|_{\beta_0=0} = \delta r_\alpha - 2c_{0,\alpha\alpha}\alpha_0 = \delta r_\alpha - 0 > 0.$
- $\frac{\partial U_G}{\partial \beta_0}\Big|_{\alpha_0=0} = \delta r_\beta - 2c_{0,\beta\beta}\beta_0 = \delta r_\beta - 0 > 0.$

Here we prove the two conditions for U_D:

1. $U_D(\alpha_i = 0, \beta_i = 0) = (1-\delta)r^T\gamma_0 - 0 \geq 0$
2. We prove this for each constraint, $\alpha_i \geq \alpha_0, \beta_i \geq \beta_0$:

- $\frac{\partial U_D}{\partial \alpha_i}\Big|_{\beta_i=\beta_0} = (1-\delta)r_\alpha - 2c_{i,\alpha\alpha}(\alpha_1 - \alpha_0) = (1-\delta)r_\alpha > 0.$
- $\frac{\partial U_G}{\partial \beta_0}\Big|_{\alpha_i=\alpha_0} = (1-\delta)r_\beta - 2c_{i,\beta\beta}(\beta_i - \beta_0) = \delta r_\beta - 0 > 0.$

□

Proving the Backfiring Result

Below we prove the Theorem 6.1.

Theorem 6.1. *Given an AI regulation game with quadratic costs. If both players' cost interactions meet the following conditions:*

$$c_{p,\alpha\beta} < \min\left(\sqrt{c_{p,\alpha\alpha}c_{p,\beta\beta}}, \frac{c_{p,\alpha\alpha}r_\beta}{r_\alpha}, \frac{c_{p,\beta\beta}r_\alpha}{r_\beta}\right),$$

then there exists an $\epsilon > 0$ such that the regulatory regime $\theta_G = 0, \theta_D = \beta_0^A - \epsilon$ backfires.

Proof. Assume $\theta_G = 0$ for the entire proof. By Remark 4.3, we're given that the players commit to their unconstrained strategy in equilibrium. These were solved in Propositions 4.4 and 4.5. Thus we have the following player's strategies under no regulation for this setting:

$$\gamma_0^A = \frac{\delta}{2}C_0^{-1}r, \quad \gamma_1^A = \frac{1-\delta}{2}C_1^{-1}r. \qquad [1]$$

Our strategy is to show that G's unconstrained, no-regulation optimum becomes dominated in the presence of regulation targeting D, which we choose to be arbitrarily close to β_1^A.

Notation. Before we proceed, we introduce some additional notation. Define the set S to be all feasible pairs of strategies (γ_0, γ_1). 'Feasible' here means those strategies which leave both G and D with non-negative utility. We use the subscript S_{θ_D} to track the particular regulatory threshold. The feasible pairs of strategies in the unregulated game is given by S_0, and the feasible pairs of strategies in a game with threshold $\theta_D = 1.5$ is denoted $S_{1.5}$. We may refer to the unregulated game with the superscript A (for anarchy), e.g. β_1^A refers to the unregulated safety level. Observe that any set of tuples S_θ can be separated into two mutually exclusive and collectively exhaustive sets:

- S_θ^{MC} (for minimally compliant) is the set of all tuples where D's best response has safety $\beta_1^* = \theta_D$.
- S_θ^{C} (for contribute) is the set of all tuples where D's best response has safety $\beta_1^* > \theta_D$.

Now, we provide a sequence of lemmas, with the purpose of establishing the intuition that *all we must do is find some $\epsilon > 0$ and some strategy $\beta_0^R \neq \beta_0^A$ such that G prefers β_0^R to β_0^A and D minimally complies.*

Lemma 3. *For any threshold $\theta_D > 0$, $S_\theta^C \subset S_0$.*

Proof. $S_0 = S_0^{\text{MC}} \cup S_0^{\text{C}} = S_0^{\text{MC}} \cup \left(\bigcup_{t=0}^{\infty} S_t^{\text{C}}\right) \supset S_\theta^{\text{C}}$. □

Lemma 4. *If $\theta_D \geq \beta_1^A$, backfiring is impossible.*

Proof. Assume for contradiction that $\theta_D^* \geq \beta_1^A$ and backfiring occurs. Backfiring would imply $\beta_1(\theta_D = \theta_D^*) < \beta_1(\theta_D = 0) = \beta_1^A$. However, this would violate the regulation, which we're given is greater than β_1^A. Hence we've already established the contradiction. □

Lemma 5. *Given a threshold θ, backfiring can occur only if the strategies $(\gamma_0, \gamma_1) \in S_{\theta_D}^{MC}$.*

Proof. We have established $S_{\theta_D} = S_{\theta_D}^{\text{MC}} \cup S_{\theta_D}^{\text{C}}$, so the proof will show that the strategies in S_θ^{C} can never exhibit backfiring. This would imply, if backfiring occurs over the feasible set of strategies S_θ, it is only possible for strategies in S_θ^{MC}. The proof proceeds, first for all values $\theta_D \geq \beta_1^A$, and then for all values $\theta < \beta_1^A$.

- For $\theta_D \geq \beta_1^A$, backfiring is impossible generally, as established in Lemma 4.

- For $\theta_D < \beta_1^A$, start by observing that the anarchy solution (γ_0^A, γ_1^A) is always feasible. This solution is the strategy tuple that maximizes the utility of G over S_0. Lemma 3 tells us that this set, S_0, contains all sets of regulated strategies where the players contribute: $S_{\theta_D}^{\text{contribute}} \subset S_0$. Thus: $(\gamma_0^A, \gamma_1^A) := \sup_{U_G} S_0 \succeq_G S_0 \supset S_{\theta_D}^{\text{contribute}} \rightarrow (\gamma_0^A, \gamma_1^A) \succeq_G S_{\theta_D}^{\text{C}}$. Thus the anarchy solution is feasible and dominates all strategies in $S_{\theta_D}^{\text{contribute}}$.

This completes the proof, and demonstrates that if backfiring is ever to occur, it will exhibit strategies that are *minimally compliant* with the regulation. □

Backfiring is a regulation yielding lower safety than β_0^A. The claims above state that backfiring cannot occur if $\theta_D > \beta_1^A$ and can only occur if the domain specialist minimally complies. As an immediate corollary, we can claim that backfiring occurs *if and only if* there is a regulation $\theta_D < \beta_1^A$ such that the strategies $(\gamma_0(\theta_D), \gamma_1(\theta_D)) \in S_{\theta_D}^{MC}$.

Lemma 6. *For a given regulation $\theta_D < \beta_1^A$ in our setting, if G prefers any minimally compliant strategy γ_0' to γ_0^A, then G's optimal strategy $\gamma_0^* \in S_{\theta_D}^{MC}$ and the regulation backfires.*

Proof. We're given γ_0^A is optimal over S_0. By Lemma 3, $S_{\theta_D}^C \subset S_0$. Since $\theta_D < \beta_1^A$, γ_0^A remains feasible. The only new strategies available to G are those in $S_{\theta_D}^{MC}$. Thus, if we denote utility-domination using $\succ$, we have $\gamma_0' \succ \gamma_0^A \succeq g \forall g \in S_{\theta_D}^C$. This implies G's optimal strategy γ_0^* is either γ_0' or otherwise belongs to $S_{\theta_D}^{MC}$. □

Thus our task is to find some regulation θ_D and some strategy γ_0' such that $U_G(\gamma_0') > U_G(\gamma_0^A)$.

Lemma 7. *For small $\epsilon > 0$, if the given conditions are met, the following G strategy dominates no regulation:*

$$\gamma_0' = \begin{bmatrix} \frac{\delta r_\alpha}{2c_{0,\alpha\alpha}} \left(\beta_0^A - 2\epsilon\right) \\ \beta_0^A - 2\epsilon \end{bmatrix}$$

Proof. Equation 1 give us G and D's strategies under no regulation. Given G's candidate strategy stated in the Lemma, we compute D's best response. Observe this must be a minimally-compliant best response, because G's strategy was constructed to be a difference $\beta_0^A + \epsilon$ from D's regulatory floor. Thus, by Proposition 4.4, we have:

$$\gamma_1' = \begin{bmatrix} \alpha_0' + \frac{(1-\delta)}{2c_{1\alpha\alpha}} - \frac{c_{1,\alpha\beta}}{c_{1\alpha\alpha}}\theta_D \\ \theta_D \end{bmatrix}$$

We compare G's utility in the two scenarios:

1. $(\gamma_0', \gamma_1') \rightarrow U_G' = \delta\left(r_\alpha \alpha_1' + r_\beta \beta_1'\right) - c_{0,\alpha\alpha}(\alpha_0')^2 - 2c_{0,\alpha\beta}\alpha_0'\beta_0' - c_{0,\beta\beta}(\beta_0')^2$
2. $(\gamma_1^A, \gamma_1^A) \rightarrow U_G^A = \delta\left(r_\alpha \alpha_1^A + r_\beta \beta_1^A\right) - c_{0,\alpha\alpha}(\alpha_0^A)^2 - 2c_{0,\alpha\beta}\alpha_0^A\beta_0^A - c_{0,\beta\beta}(\beta_0^A)^2$

We compute the difference $\Delta U_G = U_G' - U_G^A$. We expand both terms and take the limit as $\epsilon \searrow 0$ to get the following:

$$\lim_{\epsilon \searrow 0} \Delta U_G = \frac{\delta(1-\delta) r_\beta}{2c_{1\beta\beta}} \left(r_\beta - \frac{c_{1,\alpha\beta}}{c_{1,\alpha\alpha}} r_\alpha \right)$$

A sufficient condition for this quantity being positive is stated below. The reason is all terms outside the parentheses are given as positive.

$$r_\beta > \frac{c_{1,\alpha\beta}}{c_{1,\alpha\alpha}} r_\alpha.$$

Notice the above condition is given as it is one of the conditions in remark 4.3.* □

Thus, we have shown that for small positive ϵ, the generalist prefers the backfiring strategy to the unconstrained optimum γ_0^A. By Lemma 6, the optimal regulated strategy is an element in $S_{\theta_D}^{MC}$ and the regulation backfires. □

Proof of the mutualism result

Here we prove Theorem 6.2, that for a swath of games there exists a set of regulations that mutually improve the player's utilities.

Theorem 6.2. *Given a two-player AI regulation game with quadratic costs. If both players meet the following conditions:*

$$|c_{p,\alpha\beta}| < \min\left(\sqrt{c_{p,\alpha\alpha} c_{p,\beta\beta}}, \frac{c_{p,\alpha\alpha} r_\beta}{r_\alpha}, \frac{c_{p,\beta\beta} r_\alpha}{r_\beta}\right),$$

then there exists an $\epsilon > 0$ such that the regulatory regime $\theta_G = \beta_0^A + \epsilon, \theta_D = \beta_1^A + 2\epsilon$ mutually improves both players' utilities.

*This is also the condition for having a non-zero safety investment when costs are convex, and intuitively, backfiring is impossible when safety investment is zero.

Proof. Observe that we only have to provide a single instance of regulation that does better than the unregulated optimal γ_0^A, γ_1^A to show that there exists a Pareto improvement effect of regulation. We consider the following minimal-compliance strategies (using Proposition 4.4 and 4.5):

$$\gamma_0' = \begin{bmatrix} \frac{\delta r_\alpha}{2c_{0,\alpha\alpha}} - \frac{c_{0,\alpha\beta}}{c_{0,\alpha\alpha}}\theta_G \\ \theta_G \end{bmatrix}, \gamma_1' = \begin{bmatrix} \alpha_0 + \frac{(1-\delta)r_\alpha}{2c_{1,\alpha\alpha}} - \frac{c_{1,\alpha\beta}}{c_1\alpha\alpha}\theta_D \\ \theta_D \end{bmatrix}.$$

Observe these are feasible because they are compliant and, for small ϵ, the performance investment is positive.

Lemma 8. *For the specified conditions, $U_G(\gamma_0', \gamma_1') > U_G(\gamma_0^A, \gamma_1^A)$*

Proof. Start by computing the change in the generalist's performance and safety investments between these strategies. The change in safety investment is simply $\Delta\beta_0 = \theta_G - \beta_0^A = \epsilon$. The change in performance investment is given by:

$$\begin{aligned}
\Delta\alpha_0 = \quad & \frac{\delta r_\alpha}{2c_{0,\alpha\alpha}} - \frac{c_{0,\alpha\beta}}{c_{0,\alpha\alpha}}\left(\frac{\delta}{2\det C_0}(-c_{0,\alpha\beta}r_\alpha + c_{0,\alpha\alpha}r_\beta + \epsilon)\right) - \frac{\delta}{2\det C_0}\left(c_{0\beta\beta}r_\alpha - c_{0\alpha\beta}r_\beta\right) \\
= \quad & \frac{\delta r_\alpha}{2c_{0,\alpha\alpha}} + \frac{c_{0,\alpha\beta}^2}{c_{0,\alpha\alpha}}\frac{\delta r_\alpha}{2\det C_0} - c_{0,\alpha\beta}\frac{\delta}{2\det C_0}r_\beta + \frac{c_{0,\alpha\beta}}{c_{0,\alpha\alpha}}\epsilon - \frac{\delta}{2\det C_0}c_{0,\beta\beta}r_\alpha + c_{0,\alpha\beta}\frac{\delta}{2\det C_0}r_\beta \\
= \quad & \left(\frac{\delta}{2c_{0,\alpha\alpha}} + \frac{c_{0,\alpha\beta}^2\delta}{c_{0,\alpha\alpha}2\det C_0} - \frac{\delta c_{0,\beta\beta}}{2\det C_0}\right) r_\alpha + \frac{c_{0,\alpha\beta}}{c_{0,\alpha\alpha}}\epsilon \\
= \quad & \frac{\delta r_\alpha}{2}\left(\frac{1}{c_{0,\alpha\alpha}} + \frac{c_{0,\alpha\beta}^2}{c_{0,\alpha\alpha}(c_{0,\alpha\alpha}c_{0,\beta\beta} - c_{0,\alpha\beta}^2)} - \frac{c_{0,\beta\beta}}{c_{0,\alpha\alpha}}\right) + \frac{c_{0,\alpha\beta}}{c_{0,\alpha\alpha}}\epsilon \\
= \quad & \frac{\delta r_\alpha}{2}\left(\frac{\det C_0 + c_{\alpha\beta}^2 - c_{0,\alpha\alpha}c_{0,\beta\beta}}{c_{0,\alpha\alpha}\det C_0}\right) + \frac{c_{0,\alpha\beta}}{c_{0,\alpha\alpha}}\epsilon \\
= \quad & \frac{c_{0,\alpha\beta}}{c_{0,\alpha\alpha}}\epsilon.
\end{aligned}$$

By the same logic, we solve for the change in the players' strategies. First, $\Delta\beta_1 = \beta_1' - \beta_1^A = \beta_1^A + 2\epsilon - \beta_1^A = 2\epsilon$. The change in performance is given by:

$$\begin{aligned}
\Delta\alpha_1 = \quad & \alpha_1' - \alpha_1^A \\
= \quad & \left[\alpha_0' + \frac{(1-\delta)r_\alpha}{2c_{1,\alpha\alpha}} - \frac{c_{1,\alpha\beta}}{c_{1,\alpha\alpha}}(\theta_D - \beta_0')\right] - \left[\alpha_0^A + \frac{(1-\delta)}{2\det C_1}(c_{1,\beta\beta}r_\alpha - c_{1,\alpha\beta}r_\beta)\right] \\
= \quad & \Delta\alpha_0 + \frac{(1-\delta)r_\alpha}{2c_{1,\alpha\alpha}} - \frac{c_{1,\alpha\beta}}{c_{1,\alpha\alpha}}\left(\frac{(1-\delta)}{2\det C_1}(-c_{1,\alpha\beta}r_\alpha + c_{1,\alpha\alpha}r_\beta) + \epsilon\right) - \frac{(1-\delta)}{2\det C_1}(c_{1,\beta\beta}r_\alpha - c_{1,\alpha\beta}r_\beta) \\
= \quad & \frac{c_{0,\alpha\beta}}{c_{0,\alpha\alpha}}\epsilon + \frac{(1-\delta)r_\alpha}{2c_{1,\alpha\alpha}} - \frac{c_{1,\alpha\beta}}{c_{1,\alpha\alpha}}\left(\frac{(1-\delta)}{2\det C_1}(-c_{1,\alpha\beta}r_\alpha + c_{1,\alpha\alpha}r_\beta) + \epsilon\right) - \frac{(1-\delta)}{2\det C_1}(c_{1,\beta\beta}r_\alpha - c_{1,\alpha\beta}r_\beta) \\
= \quad & \epsilon\left(\frac{c_{0,\alpha\beta}}{c_{0,\alpha\alpha}} - \frac{c_{1,\alpha\beta}}{c_{1,\alpha\alpha}}\right).
\end{aligned}$$

The change in G's cost is given by:

$$\begin{aligned}
\Delta(\text{G's cost}) = \quad & \begin{bmatrix}\Delta\alpha_0 \\ \Delta\beta_0\end{bmatrix}^T C_0 \begin{bmatrix}\Delta\alpha_0 \\ \Delta\beta_0\end{bmatrix} \\
= \quad & c_{0,\alpha\alpha}\left(\frac{c_{0,\alpha\beta}}{c_{0,\alpha\alpha}}\right)^2\epsilon^2 + 2\left(\frac{c_{0,\alpha\beta}}{c_{0,\alpha\alpha}}\epsilon\right)\epsilon + c_{0\beta\beta}\epsilon^2
\end{aligned}$$

Notice these are all ϵ^2 terms, meaning as ϵ is brought to very small positive values, they approach zero at an exponential rate. The contribution to G's revenue is given by:

$$\begin{aligned}
\Delta(\text{G's revenue}) = \quad & \delta(r_\alpha\Delta\alpha_1 + r_\beta\Delta\beta_1) \\
= \quad & \delta\left(r_\alpha\epsilon\left(\frac{c_{0,\alpha\beta}}{c_{0,\alpha\alpha}} - \frac{c_{1,\alpha\beta}}{c_{1,\alpha\alpha}}\right) + r_\beta 2\epsilon\right)
\end{aligned}$$

Notice these are terms of ϵ, whereas the cost effects are solely terms of ϵ^2. Therefore, for sufficiently small ϵ, we say:

$$\lim_{\epsilon\searrow 0}\Delta U_G = \delta\left(r_\alpha\epsilon\left(\frac{c_{0,\alpha\beta}}{c_{0,\alpha\alpha}} - \frac{c_{1,\alpha\beta}}{c_{1,\alpha\alpha}} + 2r_\beta\epsilon\right)\right)$$

Using the given conditions, we know:

$$\begin{aligned}
\lim_{\epsilon\searrow 0}\Delta U_G > 0 \iff \quad & \delta\left(r_\alpha\epsilon\left(\frac{c_{0,\alpha\beta}}{c_{0,\alpha\alpha}} - \frac{c_{1,\alpha\beta}}{c_{1,\alpha\alpha}}\right) + 2r_\beta\epsilon\right) > 0 \\
\iff \quad & r_\alpha\left(\frac{c_{0,\alpha\beta}}{c_{0,\alpha\alpha}} - \frac{c_{1,\alpha\beta}}{c_{1,\alpha\alpha}}\right) + 2r_\beta > 0 \\
\iff \quad & \frac{r_\alpha c_{0,\alpha\beta}}{r_\beta c_{0,\alpha\alpha}} - \frac{r_\alpha c_{1,\alpha\beta}}{r_\beta c_{1,\alpha\alpha}} > -2.
\end{aligned}$$

Our conditions strictly bound the absolute value of both terms on the left hand side below 1, so this completes the Lemma's proof. □

Lemma 9. *For the specified conditions,* $U_D(\gamma_0', \gamma_1') > U_D(\gamma_0^A, \gamma_1^A)$

The limiting effect on D's revenue is calculated exactly the same way as above, except that the revenue expression is multiplied by $(1 - \delta)$ instead of δ.

This completes the proof, as both players are better off under the regulation. □
